# Sterol regulation in *Komagataella phaffii*: Identification of sterol regulatory element binding proteins and their activation pathway

**DOI:** 10.1016/j.jbc.2025.110579

**Published:** 2025-08-08

**Authors:** Simon Arhar, Melanie Merl, Odysseas Pantelakis, Franziska Gruber, Paula Berzak, Heimo Wolinski, Anita Emmerstorfer-Augustin

**Affiliations:** 1 Institute of Molecular Biotechnology, Graz University of Technology, NAWI Graz, Graz, Austria; 2 Institute of Molecular Biosciences, University of Graz, Graz, Austria; 3 BioHealth-Graz, Graz, Austria; 4 Austrian Centre of Industrial Biotechnology, acib GmbH, Graz, Austria; 5 BioTechMed-Graz, Graz, Austria

**Keywords:** sterol, yeast metabolism, yeast transcription, Komagataella phaffii, sterol biosynthesis

## Abstract

Sterol biosynthesis in eukaryotes is commonly regulated by Sterol Regulatory Element Binding Proteins (SREBPs), membrane-bound transcription factors activated by proteolytic cleavage in response to sterol levels. While extensively studied in mammals, SREBP function and regulation in fungi remain less understood. Here, we identify and characterize two SREBP homologs, Hms1-1 and Hms1-2, in the methylotrophic yeast *Komagataella phaffii*. Transcriptional and physiological analyses reveal that Hms1-1 and Hms1-2, together with Upc2, form a central regulatory hub controlling sterol-responsive gene expression. Despite largely unchanged total sterol levels, the double deletion mutant (*hms1-1Δ hms1-2Δ*) exhibits altered expression of sterol biosynthesis genes and increased sensitivity to terbinafine, indicating a sterol-responsive role for SREBPs. Remarkably, *K. phaffii* retains both Upc2 and SREBP pathways, an unusual dual regulatory system in fungi that highlights evolutionary plasticity. Functional assays demonstrate that overexpression of either *HMS1-1* or *HMS1-2* rescues the lethality of *upc2Δ* mutants, revealing redundancy and flexibility in sterol regulation. We show that proteolytic activation of these homologs depends on Scp, a functional analog of mammalian Scap, and the Dsc E3 ligase complex. Comparative analyses identify distinct roles: Hms1-2 acts as the primary, Scp-dependent activator, whereas Hms1-1 is constitutively processed, less Scp-dependent, and likely regulated by post-translational sumoylation. These findings establish *K. phaffii* as a genetically tractable model for studying SREBP signaling and provide new insights into the evolution and complexity of fungal sterol homeostasis.

Lipid regulation in mammalian cells is primarily controlled by sterol regulatory element-binding proteins (SREBPs), a family of transcription factors anchored in the endoplasmic reticulum (ER) membrane (1). SREBPs regulate the expression of over 30 genes involved in cholesterol, fatty acid, triglyceride, and phospholipid metabolism in higher eucaryotes (2, 3, 4). This regulatory mechanism has been found to be evolutionarily conserved across animals and fungi. In 2005, the first fungal orthologs, Sre1 and Sre2, were identified in the fission yeast *Schizosaccharomyces pombe* (5). Like mammalian SREBPs, Sre1 undergoes sterol-dependent proteolytic activation and regulates genes essential for sterol homeostasis. Additionally, Sre1 plays a pivotal role in the transcriptional response to hypoxia, supporting growth under low-oxygen conditions (6). Subsequent studies identified and characterized SREBPs in human fungal pathogens, such as *Candida albicans* (7), *Cryptococcus neoformans* (8), and *Aspergillus fumigatus* (9). The presence of SREBPs in both the Basidiomycota (*C. neoformans*) and Ascomycota (*S. pombe*, *A. fumigatus, and C. albicans*) phyla underscores the widespread conservation of this regulatory pathway across the fungal kingdom. However, in Saccharomycotina, a subgroup of Ascomycota that includes *Saccharomyces cerevisiae* and *C. albicans*, sterol regulation is controlled by Upc2, an unrelated transcription factor with a Gal4-type zinc finger (7, 10). Within this subgroup, evolution introduced a unique shift in regulatory mechanisms. While these yeasts still produce SREBPs, their role in sterol regulation appears to have been lost, with their ancestral function in filamentous growth retained (7, 11). The evolutionary transition from SREBPs to Upc2 was further investigated in the deep-branching species *Yarrowia lipolytica*, the only yeast known so far to harbor a functional ortholog of Upc2 and a sterol metabolism regulating SREBP variant (Sre1) (12). This transition seems to have occurred in two distinct steps: First, Upc2 emerged in an ancestor of Saccharomycotina, taking over sterol regulatory functions. Subsequently, SREBPs lost their role in sterol regulation but retained their involvement in filamentation.

In mammalian cells, the activity of SREBPs is tightly regulated by a sterol-dependent feedback mechanism, ensuring activation under low sterol conditions and inactivation when sterol levels are high (reviewed by (13)). Upon activation, SREBPs are escorted by Scap, a sterol-sensing protein that facilitates their transport from the endoplasmic reticulum (ER) to the Golgi apparatus under low sterol conditions (2). At the Golgi, SREBPs undergo sequential proteolytic processing by two membrane-bound proteases. The first step involves Site-1 protease, a serine protease of the subtilisin family (14). The second step is carried out by Site-2 protease, a Zn^2+^ metalloprotease (15). This processing releases the N-terminal, active fragments of SREBPs, which then translocate to the nucleus to activate the transcription of sterol-regulated target genes (14, 15). When sterol levels rise, cholesterol accumulates in ER membranes, and by binding to the Insig membrane proteins, the Scap/SREBP complex is retained in the ER. This retention blocks the proteolytic activation of SREBPs, halting their ability to induce the expression of target genes involved in sterol and lipid metabolism. This feedback loop ensures tight homeostatic control of lipid levels (13).

A similar mechanism for SREBP activation has been described in *S. pombe*, where a Scap-like protein called Scp1 functions as a sterol sensor and trafficking mediator for the SREBP homolog Sre1 (16). Scp1 facilitates the transport of Sre1 from the endoplasmic reticulum (ER) to the Golgi apparatus under conditions of low oxygen or sterol depletion, enabling its activation. However, unlike the mammalian system, where Insig proteins play a central role, Scp1 has a dual function in *S. pombe*, mediating both the sterol level dependent ER retention and release of Sre1 independent of any Insig ortholog (5). Similar to the mammalian system, transport to the Golgi is necessary for Sre1 to undergo proteolytic processing. However instead of two distinct proteases, in *S. pombe*, Sre1 and its paralog Sre2 require the Golgi Dsc E3 ligase complex and the proteasome for activation - a mechanism that distinguishes it from mammalian cells (6, 17). This processing releases the N-terminal, active fragments of Sre1 or Sre2, which then relocate to the nucleus to regulate the expression of genes involved in sterol metabolism and hypoxic adaptation (16). While the SREBP activation mechanism in *S. pombe* closely resembles that of mammals, it has been adapted to meet the specific physiological and environmental needs of yeast. Interestingly, Scap homologs or proteolytic activation of SREBPs are missing in many Saccharomycotina yeasts, such as *S. cerevisiae* or *C. albicans*, underscoring the divergence in sterol regulatory mechanisms across different fungal kingdoms.

In mammalian cells, SREBP-2 activity is additionally fine-tuned by sumoylation at lysine 464 (K464), which recruits a co-repressor complex including histone deacetylase 3 (HDAC3), thereby repressing its target genes such as HMG-CoA synthase and the LDL receptor (18). This modification is dynamically regulated by phosphorylation: ERK1/2-mediated phosphorylation at serine 455 (S455), triggered by mitogenic signals like IGF-1, inhibits sumoylation and enhances SREBP-2 transcriptional activity (19). This phosphorylation-sumoylation interplay functions as a molecular switch, allowing cells to upregulate lipid biosynthesis under sterol-depleted or growth-promoting conditions. Such post-translational regulation is critical for aligning lipid metabolism with cellular demands.

In contrast to the complex nature of the SREBP activation pathway, Upc2, the main transcriptional regulator of sterol metabolism in Saccharomycotina, functions as a relatively simple, all-in-one system for modulating intracellular sterol levels. In its inactive state, Upc2 is bound to ergosterol and the chaperone Hsp90 *via* its C-terminal domain, which prevents its nuclear import. Upon sterol depletion, loss of the ergosterol ligand induces a conformational change that exposes the N-terminal nuclear localization signal, allowing Upc2 to translocate into the nucleus (20, 21). There, it activates the transcription of genes involved in ergosterol biosynthesis, sterol uptake, and the cellular response to hypoxia (22). Notably, Upc2 exerts its transcriptional activity *via* a zinc finger domain that binds to DNA motifs similar to the sterol regulatory elements (SREs) recognized by mammalian SREBPs (23).

In this study, we report the discovery of a functional SREBP pathway in the methylotrophic yeast *Komagataella phaffii*, a highly divergent member of the Saccharomycotina and an increasingly important alternative yeast model organism (24). Similar to *Y*. *lipolytica*, *K. phaffii* harbors readily identifiable orthologs of both SREBP and Upc2. While Upc2 serves as the primary regulator of genes involved in sterol biosynthesis, consistent with its well-established role across Saccharomycotina, *K. phaffii* retains a fully functional SREBP pathway. Remarkably, this pathway is regulated in a manner reminiscent of its mammalian counterpart and can partially compensate for the loss of Upc2. We identified roles for Scp and Dsc1 in the proteolytic processing of the SREBP homologs Hms1-1 and Hms1-2. Finally, we provide the first evidence that Hms1-1 likely undergoes post-translational sumoylation, suggesting additional layers of regulation.

## Results

### The *K. phaffii* genome harbors two homologous SREBP genes, *HMS1-1* and *HMS1-2*

**Figure 1:**
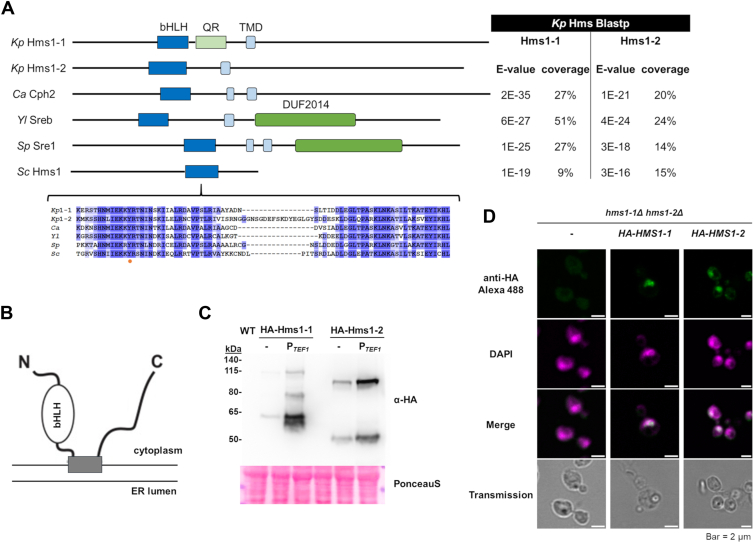
**Identification and phylogenetic analysis of Hms1-1 and Hms1-2, two homologous SREBPs in *K. phaffii*.***A*, Hms proteins were classified as SREBP-like through a BLASTp search, with a focus on the conserved basic helix-loop-helix domain. This domain includes the SREBP-specific tyrosine residue (highlighted with an orange dot) critical for interaction with the genomic sterol regulatory elements, which is conserved between *K. phaffii* Hms proteins and the top BLASTp hits. Further analysis of the amino acid sequence using CCTOP and PFAM revealed a similar domain organization in the Hms proteins. Key domains include: bHLH: Basic helix-loop-helix domain, QR: asparagine rich domain, TMD: Transmembrane domain, DUF: Domain of unknown function. *B*, predicted topology of Hms1-1 and Hms1-2. Transmembrane topology predictions were performed using Deep TMHMM (27). Both Hms1-1 and Hms1-2 are predicted to contain a single transmembrane segment, spanning amino acids 400 to 421 in Hms1-1, and 344 to 366 in Hms1-2, with both the N- and C-termini oriented toward the cytosol. The N-terminal region harbors a bHLH DNA-binding domain similar to those found in mammalian SREBPs and the Sre1 protein of *S. pombe*. *C*, a CBS7435 *his4*Δ strain expressing *HA-HMS1-1* from the endogenous *HMS1-1* locus (yFG049), *HA-HMS1-2* from the endogenous *HMS1-2* locus (yFG059) and strains *hms1-1*Δ *hms1-2*Δ expressing *HA-HMS1-1* (yFG071) and *HA-HMS1-2* (yFG073) under the strong *TEF1* promoter from the *his4*Δ locus, were cultivated in MD-his media to middle exponential phase at 28 °C, harvested, lysed, and the proteins were extracted, resolved by SDS–PAGE, and analyzed by immunoblotting with anti-HA antibody, as described under Materials and Methods. Loading control, PonceauS detected on the same blots prior to blocking and immunodetection. MW, marker proteins (kDa). Expected molecular weights: Hms1-1 full length (109 kDa); Hms1-2 full length (84 kDa). *D*, the same strains as in (C) (yFG071 and yFG073) were grown to exponential phase in MD-his media. The parental *hms1-1*Δ *hms1-2*Δ strain (yFG060) was included as background control for immunolabelling. Cellular localization of HA-tagged Hms1-1 and Hms1-2 was determined immunologically using an anti-HA IgG1 Alexa Fluor 488 conjugate16B12 (green) and DAPI staining (magenta) as described in the Materials and Methods. Representative images of each HMS variant are shown.

In a previous study, we demonstrated that the *in situ* production of cholesterol, instead of ergosterol, induces lipotoxicity by massively upregulating sterol biosynthesis in *S. cerevisiae* and *K. phaffii* (25). While this effect could partially be attributed to the overproduction and potential hyperactivation of Upc2 in *K. phaffii*, sterol levels were only reduced by 20% when *UPC2* was knocked out in the cholesterol-producing strain. To explore additional factors contributing to this regulatory imbalance, we analyzed RNA-seq data from both wild-type and cholesterol-producing *K. phaffii* strains (25). This analysis revealed a striking upregulation of two homologous, hypothetical sterol-responsive transcription factors: *HMS1-1* and *HMS1-2* (Fig. S1). Sequence analysis of Hms1-1 and Hms1-2 revealed the presence of all key regulatory domains characteristic of SREBPs: a basic helix-loop-helix (bHLH) domain containing the conserved tyrosine residue essential for binding the sterol regulatory element (SRE) DNA motif and a transmembrane domain (TMD) (Fig. 1*A*). Additionally, a glutamine-rich stretch with similarity to domains found in eukaryotic Sec23 and Sec24 homologs (26) was identified in Hms1-1 using PFAM (Superfamily entry: SSF81995). These findings support the notion that Hms1-1 and Hms1-2 are functionally analogous to mammalian SREBPs and may play a central role in the regulation of sterol biosynthesis in *K. phaffii*. Further sequence analysis applying the Deep TMHMM tool (27) confirmed the presence of a single hydrophobic portion that shows high probability for being membrane-associated and does enable the typical SREBP topology with both the amino and the carboxy terminal ends being cytosolic (Fig. 1*B*). The same was reported for *Y. lipolytica* (12), while other SREBPs, as for example those found in *C. albicans* (7) and *S. pombe* (5), often possess two transmembrane helices which also leads to cytosolic localization of both termini (Fig. 1*A*). Immunoblot analysis of N-terminally HA-tagged Hms1-1 and Hms1-2 proteins showed two distinct bands: a full-length variant of expected sizes (109 kDa for Hms1-1, and 84 kDa for Hms1-2), and the N-terminus of a processed variant (Fig. 1*C*). This effect became even clearer when *HMS1-1* and *HMS1-2* were up-regulated by the *TEF1* promoter. A comparison of band intensities from these immunoblots with transcript levels (presented as transcripts per million in (Fig. S1) revealed a strong correlation between protein abundance and transcript expression. Notably, *HMS1-2* is expressed at significantly higher levels than *HMS1-1* from their native promoters (22 and five transcripts per million, respectively). Also, we noticed that overproduction of Hms1-1 resulted in the appearance of an additional third band, situated between the two expected bands, a finding that will be further investigated later in this study. To determine the intracellular localization of Hms1-1 and Hms1-2, we performed immunofluorescence microscopy with HA-tag specific antibodies (Fig. 1*D*). Consistent with the immunoblot results (Fig. 1*C*), which indicate a significant presence of the processed N-terminal fragments, Hms1-1 and Hms1-2 predominantly exhibited nuclear localization patterns.

### Hms1-1 and Hms1-2 are membrane-bound transcription factors that regulate sterol biosynthesis

**Figure 2:**
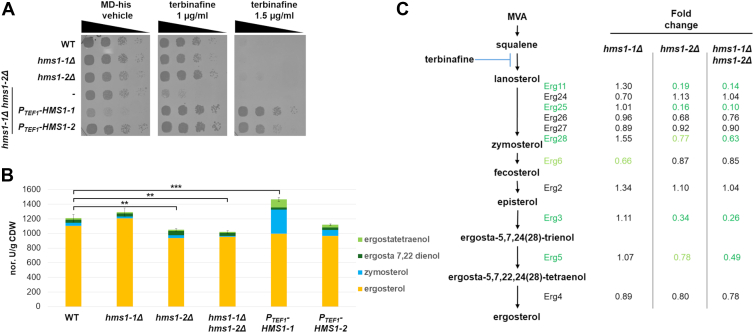
**Hms1-1 and Hms1-2 regulate sterol biosynthesis in *K. phaffii*.***A*, strains CBS7435 *his4*Δ, and isogenic derivates carrying knockouts of *hms1-1*Δ (yFG046), *hms1-2*Δ (yFG051), *hms1-1*Δ *hms1-2*Δ (yFG060), as well as *hms1-1*Δ *hms1-2*Δ strains overexpressing full-length *HMS1-1* (yFG071) or full-length *HMS1-2* (yFG073) under the *TEF1* promoter, were grown overnight in MD-his medium, diluted to an OD_600_ of 0.5, and subjected to 6-fold serial dilutions. Samples were spotted on MD-his agar plates with or without terbinafine, and growth was recorded after 48 h at 28 °C. *B*, the same strains as in (*A*) were grown overnight to an OD_600_ of three in MD-his medium, followed by sterol extraction and analysis as outlined in the Materials and Methods. Sterol measurements *via* GC-MS were normalized to an internal cholesterol standard and the cell dry weight of extracted cells (y-axis). Data of the different sterol species represent mean values from biological replicates (n = 3). Standard deviation (Stdev) of total sterols measured is indicated by error bar. Result from an unpaired two tailed *t* test for total sterol levels are indicated by asterisk (∗∗ = *p* < 0.01; ∗∗∗ = *p* < 0.001). *C*, differential expression of *ERG* genes in strains CBS7435 *his4*Δ, and isogenic derivates carrying knockouts of *hms1-1*Δ (yFG046), *hms1-2*Δ (yFG051), and *hms1-1*Δ *hms1-2*Δ (yFG060) was analyzed by qRT-PCR. Cells were grown overnight to an OD_600_ of three in MD-his media at 28 °C. Experiments were performed in triplicate, with results normalized to the 2 *K. phaffii* housekeeping genes *RSC2* and *TAF10*. Average fold changes relative to the wild type shown, represent biological replicates (n = 3). The colors of the foldchanges represent the results from a two tailed unpaired *t* test of the corresponding ΔΔCq values (light green: *p* < 0.01; dark green: *p* < 0.001) More detailed expression data are available in the Supporting Information (Fig. S2).

To investigate the roles of Hms1-1 and Hms1-2 in sterol biosynthesis, we generated single and double knockout mutants and evaluated their growth on plates supplemented with terbinafine, a potent inhibitor of sterol biosynthesis (28) (Fig. 2*A*). The wild-type strain, as well as the single mutants (*hms1-1Δ* and *hms1-2Δ*), displayed robust growth on plates containing 1 μg/ml terbinafine. In contrast, the *hms1-1Δ hms1-2Δ* double knockout strain exhibited a severe growth defect under the same conditions (Fig. 2*A*). At a higher terbinafine concentration (1.5 μg/ml), growth was completely inhibited in all strains, including the wild-type and single mutants. Interestingly, overexpression of either *HMS1-1* or *HMS1-2* driven by the strong, constitutive *TEF1* promoter in the *hms1-1Δ hms1-2Δ* double knockout strain fully restored growth, indicating that Hms1-1 and Hms1-2 have overlapping and complementary roles in sterol biosynthesis. These findings were partially consistent with measurements of total sterol levels in the cells. The single mutants (*hms1-1Δ* or *hms1-2Δ*) and the double knockout (*hms1-1Δ hms1-2Δ*) exhibited a small but significant reduction in sterol levels under normal growth conditions. Overexpression of *HMS1-1* resulted in an upregulation of sterol biosynthesis and a change in the sterol composition, which likely explains the robust rescue observed under terbinafine-induced sterol depletion (Fig. 2*B*).

To further elucidate the transcriptional regulation of sterol biosynthesis by Hms1-1 and Hms1-2, we performed qPCR analyses on *hms1-1Δ* and *hms1-2Δ* single and double knockout strains. We focused on genes involved in sterol biosynthesis downstream of lanosterol, the first sterol-like molecule in the pathway and the point of terbinafine action (29). In the *hms1-1Δ* strain, only *ERG6* was drastically downregulated. In contrast, deletion of *hms1-2* had a broader impact, resulting in the downregulation of several genes, including *ERG11*, *ERG25*, and *ERG3*. The double knockout (*hms1-1Δ hms1-2Δ*) showed an even stronger downregulation of these genes as well as *ERG5*. These results suggest that Hms1-1 and Hms1-2 play complementary and partially redundant roles in the transcriptional regulation of sterol biosynthesis, particularly through the control of key ergosterol biosynthesis (*ERG*) genes, providing insights into their potential co-dependent regulatory functions.

### Hms1-1 and Hms1-2 functionally overlap with Upc2 in the regulation of sterol biosynthesis

**Figure 3:**
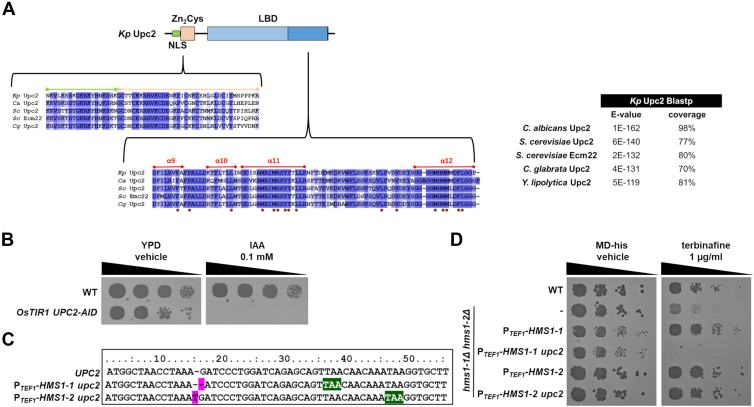
**Hms1-1 and Hms1-2 functionally overlap with Upc2 in the regulation of sterol biosynthesis.***A*, protein BLAST analysis using *S. cerevisiae* Upc2 (*Sc*Upc2) as the query revealed high similarity between the *K. phaffii* Upc2 protein and homologs from other yeast species. A schematic representation of the conserved domain organization in *K. phaffii* Upc2 is shown, including the nuclear localization signal (NLS), Zn(II)_2_Cys_6_ DNA-binding domain, and ligand-binding domain (LBD) (20). Key residues forming the hydrophobic pocket for sterol interaction are highlighted as brown dots. *B*, *K. phaffii* strain CBS7435 *his4*Δ and an isogenic strain expressing C-terminally AID-tagged *UPC2* from its endogenous locus and *TIR* under the control of the *TEF1* promoter were grown overnight in YPD medium, diluted to an OD_600_ of 0.5, and subjected to 6-fold serial dilutions. Samples were spotted on YPD or YPD plus 0.1 mM indole-3-acetic acid (IAA) agar plates, and growth was recorded after 48 h at 28 °C. *C*, sequencing of the *UPC2* locus in strains CBS7435 *his4*Δ, *hms1-1*Δ *hms1-2*Δ *upc2*Δ P_*TEF1*_-*HMS1-1* (yMIM93), and *hms1-1*Δ *hms1-2*Δ *upc2*Δ P_*TEF1*_-*HMS1-2* (yMIM94) identified frameshift mutations and indels (*magenta*), leading to in-frame stop codons (*green*) shortly after the ATG start codon. *D*, the same strains as in (*C*), along with a *hms1-1*Δ *hms1-2*Δ double deletion strain (yFG060) and strains *hms1-1*Δ *hms1-2*Δ P_*TEF1*_-*HMS1-1* (yFG71), or *hms1-1*Δ *hms1-2*Δ P_*TEF1*_-*HMS1-2* (yFG73), were grown overnight in MD-his medium, diluted to an OD_600_ of 0.5, and subjected to 6-fold serial dilutions. Samples were spotted on MD-his or MD-his agar plates containing 1 μg/ml terbinafine. Growth was recorded after 48 h at 28 °C.

Since Upc2 has not been extensively characterized in *K. phaffii*, we performed a BLASTp search using *Sc*Upc2 from *S. cerevisiae* (systemic name YDR213W) as the query. Protein BLAST analysis of the retrieved *K. phaffii* sequence (NCBI: CAH2448785.1) revealed high similarity to Upc2 homologs from other yeast species (Fig. 3*A*). A detailed evaluation of the amino acid sequence confirmed the presence of characteristic Upc2 domains in a conserved organization (22). The sequence includes a nuclear localization signal (NLS) followed by the Zn(II)_2_Cys_6_ zinc finger motif, responsible for DNA interaction. Additionally, the ligand-binding domain (LBD) at the C-terminus exhibited high similarity to other known Upc2 homologs. Notably, amino acids forming the hydrophobic pocket required for sterol interaction (highlighted as brown dots in Fig. 3*A*) (21) were conserved, further supporting the functional similarity of the *K. phaffii* Upc2 to its homologs.

To further investigate the role of *UPC2* in *K. phaffii*, we attempted to generate loss-of-function alleles using CRISPR/Cas9-induced frameshift mutations, as previously described (25). Despite repeated efforts, deletion of *UPC2* in wild-type cells was unsuccessful, indicating that the gene is essential for viability. To validate this, we constructed a strain expressing a C-terminally auxin-induced degron (AID) tagged Upc2 and performed auxin-induced degradation plate assays (25). Auxin treatment led to pronounced growth defects and loss of viability, confirming that *UPC2* is indeed essential in *K. phaffii* (Fig. 3*B*). Interestingly, deletion of *UPC2* was possible in strains overexpressing *HMS1-1* or *HMS1-2* under the strong *TEF1* promoter (Fig. 3, *C* and *D*). On MD-his plates, overexpression of either *HMS1-1* or *HMS1-2* restored growth in the *upc2Δ* background, suggesting functional redundancy. However, when cells were spotted on terbinafine-containing media, only *HMS1-2* overexpression could rescue the *upc2Δ* phenotype, while *HMS1-1* failed to do so. These findings highlight the complementary yet distinct roles of Hms1-1 and Hms1-2 in sterol biosynthesis and underscore their capacity to partially compensate for the loss of Upc2 under specific conditions.

### *K. phaffii* Scap homolog interacts with ergosterol and binds to Hms1-1 and Hms1-2

**Figure 4:**
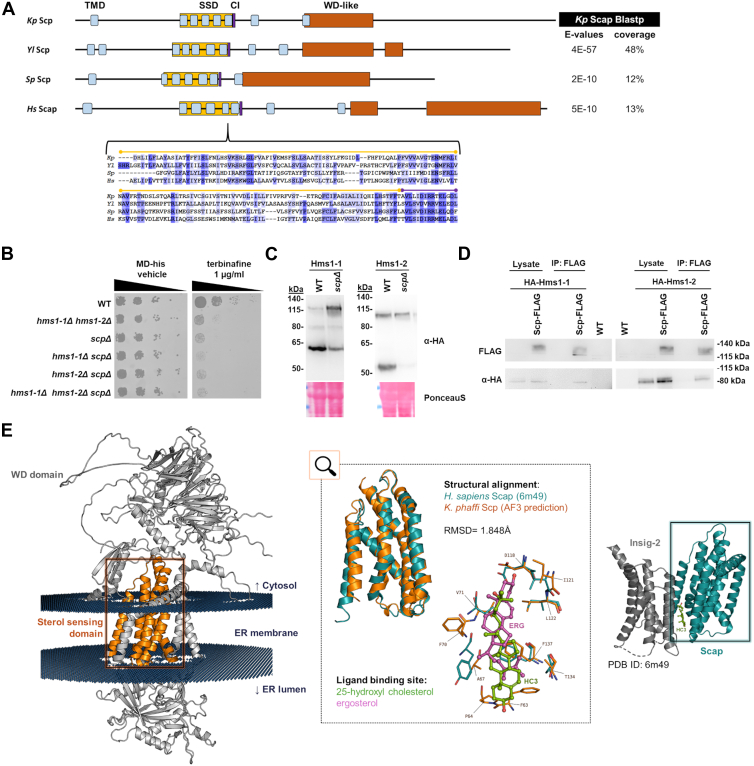
**Identification and functional characterization of a Scap homolog in *K. phaffii*.***A*, *K. phaffii* Scp (CAH2447051.1) was identified *via* BLASTp using the *S. pombe* Scp (*Sp* Scp, O43043) sequence as a query. Protein BLAST analysis revealed the best hits to known Scap homologs (*Yl*, *Y. lipolytica*; *Hs*, *Homo sapiens*), albeit with limited overall conservation. Key functional domains, including the sterol-sensing domain (SSD), a putative COPII interaction motif (CI) (39), and a WD-like domain, were predicted. CCTOP analysis of transmembrane domain organization indicated a topology comparable to characterized Scap homologs. *B*, a CBS7435 *his4*Δ strain and otherwise isogenic derivatives carrying gene knockouts of *scp*Δ (yMIM46), *hms1-1*Δ *scp*Δ (yMIM51), *hms1-2*Δ *scp*Δ (yMIM54), and *hms1-1*Δ *hms1-2*Δ *scp*Δ (yMIM57) were grown overnight in MD-his medium. Cultures were adjusted to an OD_600_ of 0.5, followed by 6-fold serial dilutions. Cell suspensions were spotted onto MD-his agar plates with or without terbinafine and incubated for 48 h at 28 °C. *C*, a CBS7435 *his4*Δ strain expressing *HA*-*HMS1-1* (yFG049), or *HA-HMS1-2* (yFG059) from their endogenous locus, or otherwise isogenic derivatives deleted for *SCP* (yMIM59, and yMIM65, respectively), were grown to middle exponential phase at 28 °C, harvested, and lysed. Proteins were extracted, resolved by SDS–PAGE, and analyzed by immunoblotting with anti-HA antibody, as described under Materials and Methods. Loading control, PonceauS detected on the same blots prior to blocking and immunodetection. MW, marker proteins (kDa). Expected molecular weights: Hms1-1 full-length (109 kDa); Hms1-2 full-length (84 kDa). *D*, pull-down assays were performed using strains expressing HA-tagged *HMS1-1* or *HMS1-2* from their endogenous loci (yFG049, yFG059), as well as strains co-expressing endogenous HA-Hms1-1 or HA-Hms1-2 with FLAG-tagged *SCP* under the control of the *GAP* promoter (yMIM127, yMIM128). CBS7435 *his4*Δ was used as a wild-type (WT) control strain. Cells were grown in MD-his, harvested, lysed, and equal amounts of protein extracts were subjected to immunoprecipitation using anti-FLAG antibodies. Total cell lysates and immunoprecipitated (IP) fractions were resolved by SDS-PAGE and analyzed by immunoblotting. Expected molecular weights: Hms1-1 full-length (109 kDa); Hms1-2 full-length (84 kDa); Scp (147 kDa). *E*, a structure of *K. phaffii* Scp predicted by AlphaFold 3 (*left panel*) was compared to an experimentally determined structure of the human Scap/Insig-2 complex (PDB: 6m49, *right panel*: turquoise and *dark grey* color, respectively). The structure of the human Scap was determined *via* cryo-electron microscopy in the presence of hydroxycholesterol HC3 (*green color*). The *K. phaffii* homolog is shown in a possible conformation within the ER membrane (Scp *light grey*, SSD domain highlighted in *orange*, ER membrane shown as crosses in *dark blue*). Structural alignment of the predicted structure of the sterol sensing domain (SSD) of *K. phaffii* Scp (*orange*) with the SSD of 6m49 (turquoise) indicates a root mean square deviation of 1.848 Å (*middle panel-top*). The predicted SSD of *K. phaffii* Scp (*orange*) with the most likely docking of ergosterol (ERG, *pink*) (*middle panel-bottom*) is shown aligned to 6m49 with its bound ligand 25-hydroxyl cholesterol (HC3, *green*) (ligands as ball and stick, residues responsible for sterol binding as lines).

To identify potential Scap-like proteins in *K. phaffii*, a BLASTp search was conducted using the *S. pombe* Scp1 (UniProt ID: O43043) sequence as a query. Analysis of the best *K. phaffii* Scp candidate (147 kDa), further indicated the highest similarities to known Scap homologs from *Y. lipolytica* (NCBI: XP_504843.4), *S. pombe* (UniProt ID: O4304) and *Homo sapiens* (UniProt ID: Q12770), though with limited overall conservation (Fig. 4*A*). Despite this, key functional domain characteristics of Scap, including the sterol sensing domain (SSD, Interpro entry: IPR000731) (30), a putative COPII interaction motif (CI) (31), and a C-terminal WD40 domain (Interpro entry: IPR001627) (32), were predicted. CCTOP analysis of transmembrane domain organization (33) revealed a topology consistent with known Scap homologs.

Growth assays revealed that the *scp*Δ single mutant, the *hms1-1*Δ *scp*Δ and *hms1-2Δ scp*Δ double mutants, as well as the *hms1-1*Δ *hms1-2*Δ *scp*Δ triple mutant, all exhibited pronounced growth defects on plates supplemented with 1 μg/ml terbinafine (Fig. 4*B*). These findings suggest a functional role for Scp in supporting the activity of Hms1-1 and Hms1-2. To explore this further, we analyzed proteolytic activation of HA-tagged Hms1-1 and Hms1-2 expressed from their endogenous loci in a *scp*Δ background. Immunoblotting revealed a marked reduction in the appearance of the lower molecular weight, proteolytically processed forms of both transcription factors (Fig. 4*C*). To test whether Scp physically interacts with Hms1-1 and Hms1-2, we performed co-immunoprecipitation assays using FLAG-tagged Scp and epitope-specific beads. Both HA-tagged Hms proteins were specifically enriched in the pull-down fractions in the presence of FLAG-Scp, supporting a direct interaction between Scp and the Hms proteins (Fig. 4*D*).

To test whether *K. phaffii* Scp directly interacts with sterols, as shown for mammalian Scap and cholesterol (34), we performed a PhotoClick cholesterol capture assay (35) using microsomal fractions enriched in Scp-FLAG. Immunoblotting of the pulled-down fraction confirmed the presence of Scp-FLAG, supporting its ability to bind sterol analogs (Fig. S3). To further explore ergosterol recognition, we generated a high-confidence AlphaFold3 (36) structural model of Scp, revealing distinct WD-like cytosolic, transmembrane, and ER-luminal domains (Figs. 4*E* and S4). Disordered loops and helices showed lower confidence and were excluded from downstream analysis. Membrane orientation was refined using the PPM server (37, 38), which guided ergosterol docking simulations to predict potential sterol-binding regions. The docking simulations were completed using the transmembrane domains of Scp, also forming the SSD, as a scaffold *via* the SwissDock server and plausible ligand orientations were further examined. As a structural analog and comparison for ligand binding, a human Scap-Insig protein complexed with hydroxycholesterol (PDB ID: 6m49, cryo-EM) was used. The two SSD domains aligned to a high degree (RMSD 1.848 Å), with the proteins sharing conserved residue segments, comprised of either identical or functionally similar amino acids. The ligand docking states for *K. phaffii* Scp were localized around three hydrophobic pockets formulated by the transmembrane helices, the most frequent of which matched closely the one occupied by hydroxycholesterol in the human analog. Interestingly, six out the 10 most energetically favorable states for ergosterol were around this cavity, with some orientations almost matching the experimentally solved cholesterol ligand (Table S1). Under closer inspection, in all ligand orientations found in this hydrophobic pocket, ergosterol was interacting with the same residues, many of which are conserved between the human and the *K. phaffii* Scap. Most of them belong to the hydrophobic amino acids, while a few polar ones are responsible for hydrogen bonding with the hydroxyl groups of the ligands.

### Posttranslational processing of Hms1-1 and Hms1-2 includes Dsc1-driven proteolytic cleavage and sumoylation

**Figure 5:**
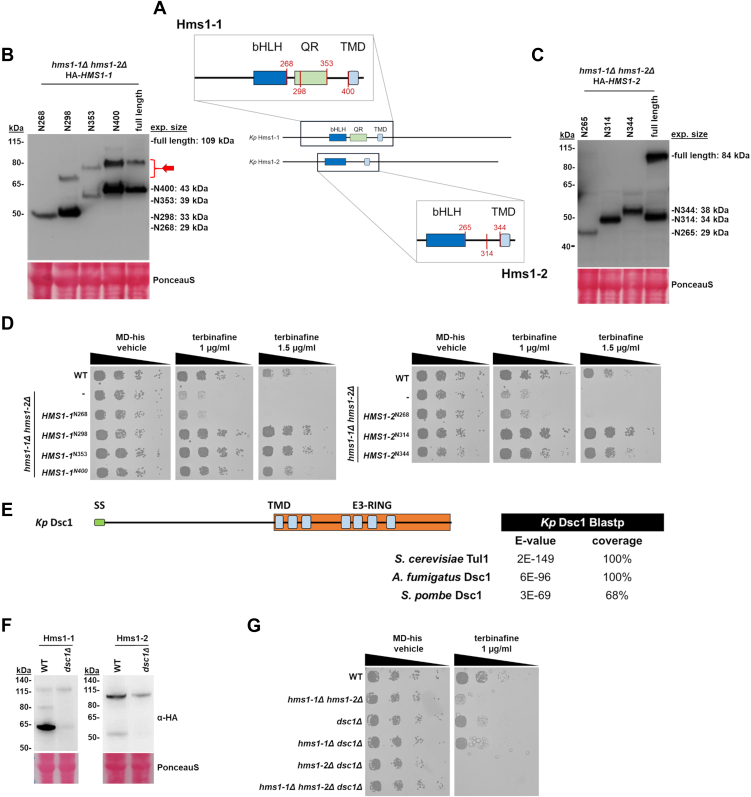
**Probing the proteolytic processing of Hms1-1 and Hms1-2.***A*, schematic overview of the C-terminal truncations (*red*) of Hms1-1 and Hms1-2 analyzed in this study. Expected molecular weights: Hms1-1 full-length (109 kDa), N400 (43 kDa), N353 (39 kDa), N298 (33 kDa), N268 (29 kDa); Hms1-2 full-length (84 kDa), N344 (38 kDa), N314 (34 kDa), N265 (29 kDa). Domain annotations: bHLH, basic helix-loop-helix; PI, protein interaction domain; TMD, predicted transmembrane domains. *B*, *hms1-1*Δ *hms1-2*Δ strains expressing *HMS1-1* (yMIM122,109 kDa), *HMS1-1*^N400^ (yMIM115), *HMS1-1*^N353^ (yMIM116), *HMS1-1*^N298^ (yMIM117) and *HMS1-1*^N268^ (yMIM113) from the *GUT1* promoter were grown on MD-his medium to middle exponential phase at 28 °C, harvested, and lysed. Proteins were extracted, resolved by SDS–PAGE, and analyzed by immunoblotting with anti-HA, as described under Materials and Methods. Loading control, PonceauS detected on the same blots prior to blocking and immunodetection. MW, marker proteins (kDa). The red arrow indicates the observed additional bands with a size shift of ∼12 kDa. *C*, *hms1-1*Δ *hms1-2*Δ strains expressing *HMS1-2* (yMIM121), *HMS1-2*^N344^ (yMIM120), *HMS1-2*^N314^ (yMIM119) and *HMS1-2*^N265^ (yMIM118) by the *GUT1* promoter were grown overnight in MD-his medium to middle exponential phase at 28 °C, harvested, and lysed. Proteins were extracted, resolved by SDS–PAGE, and analyzed by immunoblotting with anti-HA, as described under Materials and Methods. Loading control, PonceauS detected on the same blots prior to blocking and immunodetection. MW, marker proteins (kDa). *D*, strains CBS7435 *his4*Δ, an isogenic derivate carrying knockouts of *hms1-1*Δ *hms1-2*Δ (yFG060), and the same strains as under (*B*) and (*C*) were cultivated as described in Materials and Methods. Yeast cultures were adjusted to an OD_600_ of 0.5 and then samples of a set of 6-fold serial dilutions were spotted using a multiprong inoculator on agar plates containing either MD-his with vehicle alone (DMSO), 1 μg/ml, or 1.5 μg/ml of terbinafine in DMSO, and, after incubation for 48 h at 28 °C, the resulting growth was recorded. *E*, protein BLAST analysis using *K. phaffii* Dsc1 (*Kp* Dsc1) as the query revealed high similarity to homologous subunits of the E3 RING finger ubiquitin ligases complex from other yeast species. A schematic representation of the domain organization conserved between all yeast is shown, including the signal sequence for translocation into the secretory pathway (SS), seven transmembrane domains (TMD), and the carboxyterminal E3 RING finger domain (E3-RING) (20). *F*, a CBS7435 *his4*Δ strain expressing *HA*-*HMS1-1* (yFG049), or *HA-HMS1-2* (yFG059) from their endogenous locus, or otherwise isogenic derivatives deleted for *DSC1* (yMIM61, and yMIM63, respectively), were grown to middle exponential phase at 28 °C, harvested, and lysed. Proteins were extracted, resolved by SDS–PAGE, and analyzed by immunoblotting with anti-HA, as described under Materials and Methods. Loading control, PonceauS detected on the same blots prior to blocking and immunodetection. MW, marker proteins (kDa). *G*, strains CBS7435 *his4*Δ, isogenic derivates carrying knockouts of *hms1-1*Δ *hms1-2*Δ (yFG060), *dsc1*Δ (yMIM47), *hms1-1*Δ *dsc1*Δ (yMIM50), *hms1-2*Δ *dsc1*Δ (yMIM53), and *hms1-1*Δ *hms1-2*Δ *dsc1*Δ (yMIM56) were cultivated as described in Materials and Methods, and then samples of a set of 6-fold serial dilutions were spotted using a multiprong inoculator on agar plates containing either MD-his with vehicle alone (DMSO), or 1 μg/ml of terbinafine in DMSO, and, after incubation for 48 h at 28 °C, the resulting growth was recorded.

To define the minimal regions of Hms1-1 and Hms1-2 required for activity, we generated a series of N-terminal truncations and analyzed their expression, processing, and ability to restore function in *hms1-1*Δ *hms1-2*Δ double knockout strains. Truncations were designed to retain the conserved basic helix-loop-helix leucine zipper domain while progressively removing downstream regions, including predicted transmembrane domains (Fig. 5*A*). All variants were N-terminally HA-tagged and expressed from the moderately strong, constitutive *GUT1* promoter to ensure sufficient expression while avoiding potential overactivation of truncated versions. Immunoblot analysis of whole-cell lysates confirmed stable production of all Hms1-1 and Hms1-2 variants at the expected molecular weights (Fig. 5, *B* and *C*). For Hms1-1, variants N400 (43 kDa) and N353 (39 kDa) co-migrated with the cleaved form of the full-length protein, suggesting that the site of proteolytic activation lies between residues 353 and 400. Shorter variants, N298 (33 kDa) and N268 (29 kDa), migrated as expected, with N268 showing the lowest apparent molecular weight. Unexpectedly, an additional band of intermediate size (indicated by a red arrow in Fig. 5*B*) was observed in most variants except N268. Given the defined size of truncations, this band likely arises from a post-translationally modified form of the protein. To assess whether these variants were functionally active, we tested their ability to rescue the terbinafine-sensitive phenotype of the *hms1-1*Δ *hms1-2*Δ strain (Fig. 5*D*). All Hms1-1 variants, except N268, supported growth on terbinafine-containing medium, which also correlated with the presence of the intermediate band, suggesting a potential relevance for functional activation.

A similar analysis of Hms1-2 truncations revealed that processing and activity depend on sequences between residues 314 and 344 (Fig. 5*C*). Truncations N314 (34 kDa) and N344 (38 kDa) produced bands resembling the processed full-length protein and complemented terbinafine sensitivity (Fig. 5*D*). In contrast, variant N265 (29 kDa) migrated well below the size of the cleaved form and failed to restore growth, indicating a loss of activity.

To determine whether proteolytic activation of Hms1-1 and Hms1-2 depends on the Dsc ubiquitin ligase complex, we identified a homolog to the Dsc1 subunit by a BLASTp search using the *S. pombe* protein (UniProt ID: O43085) as query. A BLASTp search with the best *K. phaffii* hit (NCBI: CAH2450293; Fig. 5*E*) additionally indicated high sequence similarities to subunits of the Dsc complex from *S. cerevisiae* (UniProt ID: P36096) and *A. fumigatus* (UniProt ID: B0XQY0). A function in the Dsc ubiquitin ligase complex was further indicated by the conserved domain organization (Fig. 5*E*), including a signal sequence for translocation into the secretory pathway (SS), seven transmembrane domains (TMD), and the typical carboxy-terminal RING finger domain (PFAM Superfamily Entry IPR050731).

To verify the role of Dsc1 in Hms processing, we analyzed the band pattern of endogenously expressed HA-tagged full-length proteins in a *dsc1*Δ background. Immunoblot analysis revealed a near-complete loss of the smaller, processed variants of both Hms1-1 and Hms1-2 in the absence of Dsc1 (Fig. 5*F*). Spotting assays on terbinafine further showed that the *dsc1*Δ strain displayed growth defects (Fig. 5*G*), albeit less pronounced than those of the *hms1-1*Δ *hms1-2*Δ double deletion strain, indicating that Dsc1 is critical for the functional activation of Hms1-1 and Hms1-2.

**Figure 6:**
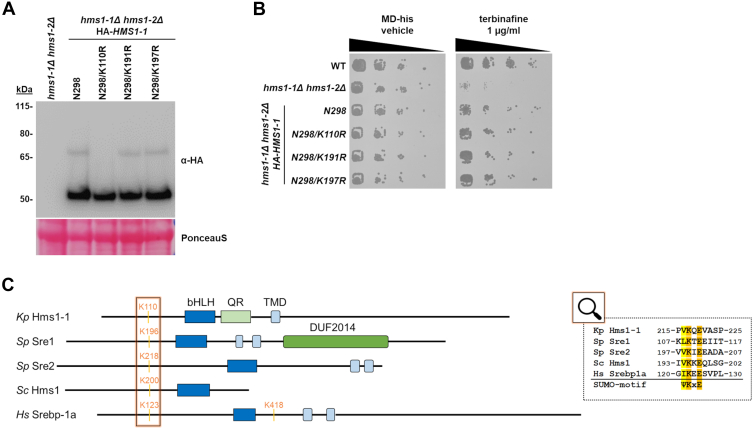
**Sumoylation as potential posttranslational modification of *K. phaffii* Hms1-1 and other fungal SREBP homologs.***A*, to analyze potential sumoylation sites, a *hms1-1*Δ *hms1-2*Δ knockout strain (yFG060), *hms1-1*Δ *hms1-2*Δ strains expressing *HMS1-1*^*N298*^*(*yMIM117) or the lysine to arginine mutants *HMS1-1*^*N298/K110R*^ (yMIM131)*, HMS1-1*^*N298/K191R*^ (yMIM132), *HMS1-1*^*N298/K171R*^ (yMIM133) from the *GUT1* promoter were grown in MD-his medium to middle exponential phase at 28 °C. Cells were harvested, proteins extracted, further resolved by SDS–PAGE, and analyzed by immunoblotting with anti-HA antibodies, as described under Materials and Methods. Loading control, PonceauS detected on the same blots prior to blocking and immunodetection. MW, marker proteins (kDa). *B*, wild-type strain CBS7435 *his4*Δ, an isogenic derivate carrying knockouts of *hms1-1*Δ *hms1-2*Δ (yFG060), and the same strains expressing truncated *HMS1-1* variants as in (E) were cultivated as described in Materials and Methods. After harvest yeast cultures were adjusted to an OD_600_ of 0.5 and then samples of 6-fold serial dilutions were spotted using a multiprong inoculator on agar plates containing either MD-his with vehicle alone (DMSO) or 1 μg/ml of terbinafine in DMSO. Shown are representative plates after incubation for 48 h at 28 °C. *C*, overview of sumoylated fungal SREBP homologs. Sumoylation of *S. cerevisiae* Hms1 and *S. pombe* Sre1 and Sre2 was reported in global SUMO proteomic approaches (43, 44, 45, 46). Similar to human SREBP-1a, all fungal SREBP homologs, including Hms1-1, exhibit a conserved sumoylation motif surrounding the modified lysines present upstream of the bHLH domain. Domains indicated include: bHLH: basic helix-loop-helix domain, QR: asparagine rich domain, TMD: transmembrane domain, DUF: domain of unknown function.

Sumoylation has been shown to play a regulatory role in the activation of mammalian SREBPs, such as SREBP-1a and SREBP-2 (40). In this study, we observed a posttranslational modification of Hms1-1 that caused a molecular weight shift consistent with the addition of a SUMO moiety (∼12 kDa) (Fig. 5*B*). This observation led us to hypothesize that the modification of Hms1-1 might be due to sumoylation. To investigate this, we used two sumoylation prediction tools, DeepSUMO (41) and GPS-SUMO (42), which identified three putative sumoylation sites within Hms1-1 that matched the consensus motif Ψ-K-X-E. Site-directed mutagenesis of these lysine residues in the C-terminally truncated variant N298 revealed that mutation of lysine 110 abolished the intermediate ∼65 kDa band (Fig. 6*A*), suggesting that this band corresponds to a sumoylated form of Hms1-1. Notably, the K110R mutant, similar to the wild-type N298 truncation, was still able to rescue the terbinafine-induced growth defect in the hms1-1Δ hms1-2Δ strain (Fig. 6*B*), indicating that while the potential sumoylation is not essential for Hms1-1 function, it may modulate activity by restricting expression of Hms1-1–regulated genes.

Although sumoylation has been previously reported for human SREBPs, it has been scarcely addressed in fungal homologs. To assess whether sumoylation might represent a more general regulatory mechanism for fungal SREBP homologs, we analyzed SUMO-proteomics datasets previously published for *S. cerevisiae* and *S*. *pombe* (Fig. 6*C*). The data revealed sumoylation of *S. cerevisiae* Hms1 at lysine 200 (43, 44), *S. pombe* Sre1 at lysine 196, and *S. pombe* Sre2 at lysine 218 (45, 46). Sequence analysis around these lysines further confirmed the presence of typical sumoylation motifs (Fig. 6*C*). Interestingly, the sumoylation sites in Hms1-1 and these fungal SREBP homologs are all located upstream of the bHLH domain, mirroring the position of sumoylation reported for human SREBP-1a. This spatial conservation may point to a conserved regulatory mechanism across species (39).

## Discussion

**Figure 7:**
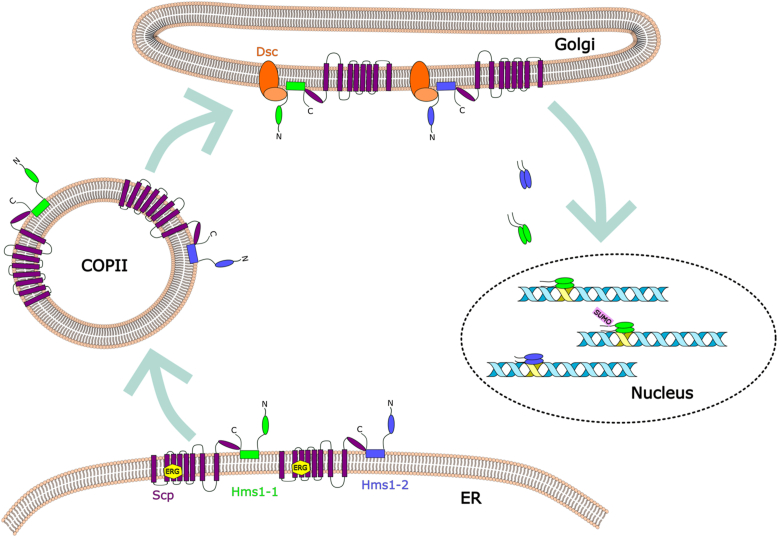
**Model of the SREBP activation pathway in *K. phaffii*.** Under conditions of sterol depletion, the transcription factors Hms1-1 and Hms1-2 are activated and trafficked to the Golgi membrane by the escort protein Scp. Presumably at the Golgi, the Dsc complex mediates their proteolytic cleavage, generating N-terminal fragments that translocate to the nucleus. There, the activated forms of Hms1-1 and Hms1-2 induce the expression of genes involved in ergosterol biosynthesis. Additionally, Hms1-1 likely undergoes sumoylation, adding a further layer of post-translational regulation to its transcriptional activity.

In this study, we identified and functionally characterized two sterol regulatory element-binding protein (SREBP) homologs, Hms1-1 and Hms1-2, in *K. phaffii*. Supporting their physiological relevance, we show that these transcription factors regulate the expression of genes involved in sterol biosynthesis. Simultaneous deletion of *HMS1-1* and *HMS1-2* alters the transcriptional profile of *ERG* genes and sensitizes cells to terbinafine, an inhibitor of early steps in sterol synthesis (Fig. 2). Although total sterol levels remain largely unaffected in the double mutant, the increased drug sensitivity under sterol-limiting conditions points to a stress-inducible regulatory role for SREBPs. Mechanistically, Hms1-1 and Hms1-2 are activated by proteolytic cleavage in a pathway requiring Scp1, a Scap-like protein, and the Dsc E3 ligase complex. While no homologs of SREBPs, Scap, or Dsc components had previously been annotated in *K. phaffii*, we identified and functionally validated the core elements of this conserved regulatory axis (Fig. 7). Together, these findings demonstrate that *K. phaffii* retains a functional SREBP signaling pathway and establish it as a genetically tractable model for investigating lipid homeostasis, with notable parallels to mammalian systems.

Importantly, this study offers new insights into the evolutionary diversification of sterol regulation in fungi. Unlike most yeasts, *K. phaffii* harbors both key transcriptional regulators of sterol metabolism: Upc2 and a functional SREBP pathway. These two systems have co-evolved within the Saccharomycotina clade but are typically not retained together. SREBP homologs have previously been identified in *S. cerevisiae* (11), *C*. *albicans* (7), *and Y. lipolytica* (12), but in *S. cerevisiae* and *C. albicans*, the sterol-dependent SREBP activation pathway is no longer functional, and Upc2 serves as the central regulator of sterol biosynthesis (47). By contrast, *Y. lipolytica* retains both a membrane-bound SREBP and a Scap homolog, alongside Upc2. Transcriptomic data suggest that both regulators contribute to sterol regulation, although detailed mechanistic insight, particularly into SREBP activation, remains limited. *Y. lipolytica* has thus far represented the only known yeast with a dual regulatory system. Our discovery of a functional SREBP pathway in *K. phaffii* identifies a second example of this dual system and provides a key link in the proposed evolutionary transition from SREBP- to Upc2-dominated regulation. Notably, overexpression of *HMS1-1* or *HMS1-2* rescues the lethality of *upc2Δ* mutants (Fig. 3), indicating that SREBPs retain the ability to drive sterol biosynthesis when sufficiently expressed. This highlights a previously unrecognized redundancy and plasticity in fungal sterol regulation. Unlike in *Y. lipolytica*, where *UPC2* is non-essential and SREBP deletions affect *ERG* gene expression only under hypoxia (12), *K. phaffii* SREBPs can fully compensate for the loss of *UPC2*, but only under the control of strong promoters. These differences likely reflect species-specific variation in SREBP expression or activation efficiency.

Mechanistically, the proteolytic activation of *K. phaffii* Hms1-1 and Hms1-2 closely resembles the established SREBP pathways in *S. pombe* and mammalian cells (5, 17). We propose that *K. phaffii* Scp functions analogously to mammalian Scap, mediating sterol-dependent trafficking of SREBPs from the endoplasmic reticulum to the Golgi apparatus, where proteolytic processing is likely initiated by the Dsc complex (Fig. 7). Despite limited sequence homology between Scp and Scap, the requirement of Scp for SREBP activation in *K. phaffii* suggests functional conservation. In addition, our identification of a Dsc1 homolog as an essential component of the proteolytic machinery supports the presence of a conserved Dsc complex, akin to its role in *S. pombe* (17). Mapping of the cleavage sites for both Hms variants further indicates that processing occurs near the transmembrane domain, consistent with findings in *S. pombe*. However, the glycine- and leucine-rich motifs critical for cleavage in *S. pombe* are only partially conserved in *K. phaffii*, suggesting potential species-specific differences in recognition or processing efficiency.

A central question emerging from our study is why *K. phaffii* has retained two homologous SREBP transcription factors, Hms1-1 and Hms1-2. While both contribute to sterol regulation, our comparative analysis reveals distinct functional features. Overexpression of either factor increases terbinafine resistance and rescues *upc2Δ* lethality, indicating functional overlap under stress. However, under standard conditions, *HMS1-2* is more highly expressed, and its deletion significantly impairs *ERG* gene expression, suggesting it serves as the primary regulator of sterol biosynthesis. Like *S. pombe* Sre1 (5), Hms1-2 depends strongly on Scp for proteolytic activation. In contrast, Hms1-1 displays several unique features not previously described for fungal SREBPs. It is expressed at lower levels, predominantly detected in its processed form even under non-stress conditions, and shows reduced dependency on Scp for activation, implying an alternative or constitutive processing mechanism. In summary, these findings suggest that Hms1-1 more closely resembles the *S. pombe* homolog Sre2, while Hms1-2 showed more similarities to Sre1 regarding function and processing (5, 17).

At the sequence level, Hms1-1 uniquely features an asparagine-rich region, a motif implicated in transcriptional regulation and protein–protein interactions (48, 49). Most notably, Hms1-1 is the first fungal SREBP indicated to undergo post-translational sumoylation, a regulatory mechanism possibly common to many fungal homologs and well established in mammals to modulate SREBP activity in response to metabolic signals (40). In our view, the findings presented in this study strongly suggest that Hms1-1 is sumoylated; however, definitive evidence, such as direct detection of the SUMO moiety on Hms1-1, is still lacking. The observed post-translational modification implies a specialized role for Hms1-1 in fine-tuning lipid regulation under specific physiological conditions. Whether this mechanism is reversible or dynamically regulated in *K. phaffii* remains an open question, but it introduces an intriguing new layer of regulation in fungal sterol control. In addition, the canonical Dsc E3 ligase complex that mediates SREBP cleavage in *S. pombe* appears to be incompletely conserved in *K. phaffii*, which lacks clear homologs of Dsc2, Dsc4, and Rbd2 (17, 50). This raises the possibility that *K. phaffii* employs an alternative proteolytic machinery or trafficking pathway for SREBP activation. Identifying the proteases involved and elucidating the activation mechanism, particularly for Hms1-1, will be crucial to fully understand sterol regulation in this yeast.

Over the past decades, *K. phaffii* has emerged as a versatile non-conventional yeast, valued both as a robust industrial production host and as a tractable model for fundamental research (24). A key advantage lies in its evolutionary position: Unlike *S. cerevisiae*, *K. phaffii* did not undergo whole-genome duplication, resulting in reduced genetic redundancy and clearer phenotypes upon single-gene deletions (51) (*e.g.*, *UPC2*). This simplicity facilitates functional genomics and regulatory studies. Additionally, *K. phaffii* exhibits physiologically relevant traits, such as respiration-based metabolism, mammalian-like lipid composition, and conserved DNA repair pathways, that enhance its relevance for biomedical applications (summarized in (24)).

Our study extends *K. phaffii*’s utility by uncovering a conserved and functional SREBP pathway, featuring proteolytic activation, drug-responsive behavior, and transcription factor redundancy. These findings reveal unexpected regulatory complexity and highlight *K. phaffii* as a powerful model for studying sterol homeostasis and lipid signaling, bridging fundamental biology and biotechnology.

## Experimental procedures

### Media and cultivation conditions

#### Media and cultivation

*K. phaffii* strains were grown in minimal defined (MD) medium supplemented with histidine (0.4%), biotin (0.0004%), glucose (2%), and yeast nitrogen base with ammonium sulfate without amino acids (13.4 g/L; Sigma-Aldrich). Cultures were incubated at 28 °C and 130 rpm in baffled shake flasks.

#### Spotting assays

*K. phaffii* strains were cultured overnight, and stationary-phase cells were diluted to an OD_600_ of 0.5. Six-fold serial dilutions were prepared, and 5 μl of each dilution was spotted onto MD-his agar plates (2% agarose) using a Steers-type multipronged inoculator. Terbinafine sensitivity was assessed on plates supplemented with one or 1.5 μg/ml terbinafine.

### Auxin-induced degradation assay

Assays for auxin-induced degradation of Upc2 were performed on YPD plates (2% glucose, 2% peptone, 1% yeast extract, 2% agarose) supplemented with 0.1 mM indole-3-acetic acid (IAA). Both terbinafine and IAA were dissolved in DMSO (1000 × stock solutions). All plates were incubated at 28 °C.

### Plasmid and strain construction

All plasmids used in this study (see Table S2) were constructed using Gibson Assembly (primers listed in Table S3) and verified by restriction analysis and Sanger sequencing. *E. coli* strains used for plasmid propagation were cultivated in LB medium at 37 °C with the appropriate antibiotic (Ampicillin 100 μg/ml, Zeocin 100 μg/ml, or Hygromycin B 200 μg/ml).

Transformation of *K. phaffii* was carried out according to the protocol by Lin-Cereghino *et al.*, 2005 (52). Positive transformants were selected on YPD agar plates containing the corresponding antibiotics (Zeocin 100 μg/ml, Hygromycin B 200 μg/ml). All strains used during the study can be found in Table S4.

Gene knockouts, AID-Strep-tagging of UPC2, and N-terminal 3 × HA-tagging of *HMS1-1* and *HMS1-2* were performed using a CRISPR/Cas9-based genome editing system (53), modified from Weninger *et al.*, 2018 (54). Target-specific protospacer sequences were introduced into the pPpHyg-Cas9 plasmid *via* PCR with appropriate primers (Table S3).

For genomic AID-Strep-tagging of *UPC2*, ∼1000 bp homologous arms were used to ensure correct integration. The tagging cassette (AID-tag with flanking homology regions) was cloned into the pPpKC2 plasmid backbone (55) prepared by *BamH*I and *Kpn*I digestion. To simplify transformation, the cassette was flanked by two *Smi*I sites for linearization. The *Oryza sativa TIR1* gene was expressed from the pPpT4-*TEF2*prom-*TIR1*-FLAG plasmid (53).

3×HA-tagging cassettes for *HMS1-1* and *HMS1-2* were generated by PCR amplification of the HA-tag sequence, with 35 to 40 bp of homology added *via* primer overhangs. For CRISPR-mediated genome editing, approximately 200 ng of circular pPpHyg-Cas9 plasmid and 400 ng of the AID-tag cassette or 20 ng of the 3×HA-tag cassette were used for transformation. For gene knockouts, 100 ng of the pCas9 plasmid targeting the gene of interest was used. Correct integration or introduction of frameshift mutations was confirmed by colony PCR and Sanger sequencing.

To overexpress *HA-HMS1-1-V5* and *HA-HMS1-2-V5*, the respective coding sequences were cloned into the pPpKC2-*his4*-*TEF1*prom plasmid backbone, linearized with *Pac*I and *Not*I. Expression cassettes were targeted to the *hIS4* locus using a corresponding CRISPR/Cas9 plasmid.

C-terminally FLAG-tagged *SCP* was expressed using the pPpT4-GAP plasmid (Näätsäri *et al.*, 2012), linearized with *EcoR*I and *Not*I. The *SCP* gene was amplified from the *K. phaffii* genome using target-specific primers (Table S3).

Additionally, full-length, truncated variants and sumoylation mutants of *HMS1-1* and *HMS1-2* were expressed using the pPpT4 plasmid under the control of the *K. phaffii GUT1* promoter (53). The plasmid backbone was prepared by *Smi*I and *Not*I digestion. The promoter and *HMS* gene fragments were amplified from genomic DNA or expression constructs. For transformation, ∼100 ng of *Smi*I-linearized plasmid was used. Correct integration was verified by colony PCR and Sanger sequencing.

PCRs for validating genomic modifications or amplifying native sequences for cloning were carried out using the Phire Plant Direct PCR Kit (Thermo Fisher Scientific) according to the manufacturer’s protocol.

### *In silico* sequence analysis

The *K. phaffii* proteins Hms1-1 and Hms1-2 were initially identified as potential SREBP-like proteins through BLASTp (NCBI) (56) searches against the SwissProt database and selected yeast species (accessed on [05.03.2024]). *K. phaffii* Upc2 was identified using *S. cerevisiae* Upc2 as a query in a BLASTp search (accessed on [06.03.2024]). The resulting *K. phaffii* candidate was further validated by reciprocal BLASTp searches against other yeast species with known Upc2 homologs. Similarly, *K. phaffii* Scp and Dsc1 homologs were identified using *S. pombe* Scp1 and Dsc1 sequences as queries (accessed on [12.03.2024]). Identified protein sequences were subsequently used to perform additional BLASTp searches against the SwissProt database to identify further homologs. Accession numbers and identifiers for all *K. phaffii* proteins and their homologs used for comparison are listed in Table S5.

Protein domains were predicted using the online tools Prosite (57) and Pfam (58). Transmembrane domains were analyzed with CCTOP (33), and membrane topology predictions for Hms1-1 and Hms1-2 were performed using DeepTMHMM (59). The nuclear localization signal (NLS) of Upc2 was predicted using NLStradamus (60), available *via* NovoPro. Potential sumoylation sites were predicted using DeepSUMO (40) and GPS-SUMO 2.0 (41). Multiple sequence alignments were created using Clustal Omega (61) and visualized with pyBoxshade (available from https://github.com/mdbaron42/pyBoxshade).

### Immunoblots

Unless stated otherwise, *K. phaffii* cultures were harvested at mid-exponential phase (OD_600_ = 3), corresponding to 4 OD_600_ units. Cells were pelleted by centrifugation at 3000×*g* for 5 min. Protein extraction was performed by resuspending the pellet in 300 μl of 1.85 M NaOH and 7.4% (v/v) β-mercaptoethanol, followed by incubation on ice for 10 min, as previously described by Horvath and Riezman (62). Proteins were precipitated by adding 300 μl of 50% (w/v) trichloroacetic acid (TCA), followed by incubation at 4 °C for 1 h. Samples were centrifuged at 16,000×*g* for 10 min, and pellets were washed once with 1 ml of ice-cold double-distilled water. The resulting pellets were resuspended in 50 μl of sample buffer (1 × NuPAGE LDS sample buffer supplemented with 0.33 M Tris base and 2% β-mercaptoethanol). After brief centrifugation (16,000×*g*, 20 s), 15 μl of the supernatant was loaded onto SDS-PAGE gels. SDS-PAGE was performed using NuPAGE 12% Bis-Tris Mini Protein Gels (1.0 mm; Thermo Fisher Scientific), followed by protein transfer to nitrocellulose membranes using a wet blotting system (NuPAGE, Thermo Fisher Scientific). Membranes were stained with Ponceau S (0.5% in TCA) to confirm protein transfer and then blocked for 1 h at room temperature either in TBST-BSA (3 g/l Tris base, 8.8 g/l NaCl, 0.003% Tween-20, pH adjusted to 7 with HCl, and 5% BSA) for HA-tag detection or in TBST supplemented with 2.5% (w/v) non-fat dry milk for FLAG-tag detection.

Primary antibody incubations were carried out overnight at 4 °C on a rocking platform. The following peroxidase-conjugated antibodies were used: anti-FLAG M2 (1:2500 dilution in TBST-milk; A8592, Sigma-Aldrich) and anti-HA (clone 6E2, 1:5000 dilution in 2.5% TBST-BSA; #2999, Cell Signaling Technology). Chemiluminescent signal detection was performed using Clarity Max Western ECL Substrate (Bio-Rad) and a G:Box (Syngene). The PageRuler Prestained Protein Ladder (Thermo Fisher Scientific) was used as a molecular weight marker. Specificity of the detection was verified by negative controls (wild-type strains).

### Immunofluorescence microscopy

Cells were cultured in 5 ml of MD-his medium at 30 °C overnight to reach stationary phase. The following day, the entire cell suspension was transferred into 25 ml of prewarmed fresh medium and incubated for 5 h at 30 °C. Subsequently, 5 ml of the middle exponential culture was treated with 30 mM EGTA (pH 7.0) and 10 μg/ml pepstatin A (Sigma-Aldrich, Inc.) for 5 min at room temperature (RT). Formaldehyde was then added to a final concentration of 3.7% in PEM buffer (pH 6.9; 100 mM PIPES, 1 mM EGTA, 1 mM MgCl_2_, 1 M sorbitol), and cells were fixed for 45 min with shaking. Cells were collected by centrifugation at 1000*g* using a tabletop centrifuge and washed three times with 1.5 ml of 0.1 M potassium phosphate-citrate buffer (pH 5.9). For cell wall digestion, 7.5 μl of 2-mercaptoethanol and 15 μl of 10 mg/ml zymolyase (20T; amsbio Inc.) in 1 M sorbitol were added to 1.5 ml of the cell suspension in 2 ml tubes and incubated at 32 °C for one to 1.5 h. Cell wall removal was monitored by bright-field microscopy. Spheroplasts were washed twice with 1.5 ml of PEM buffer and by centrifugation at low speed (25 g) for 2 min each using the same centrifuge. This centrifugation speed and time were used for all subsequent washing steps. For permeabilization, 30 μl of 1% Triton X-100 in PEM buffer was added to 1.5 ml of the spheroplast solution and incubated for 1 min at RT. Spheroplasts were then washed with 1.5 ml of PEM buffer. The pellet was resuspended in 1.5 ml of 2% BSA in PEM buffer and incubated for 2 h at RT. Afterwards, the spheroplasts were washed twice with 1 ml of PEM buffer. For labeling, 1 μl of anti-HA mouse IgG1, clone 16B12, Alexa Fluor 488 conjugate (1 mg/ml; Invitrogen, Inc.) in 0.5% BSA was added and incubated overnight at RT. Finally, the cells were washed once with 1 ml of PEM buffer. Labeled spheroplasts were stained with DAPI (20 μM) and mounted on standard microscope slides and covered with 0.17 mm coverslips for microscopy. Imaging was performed using a LEICA SP8 confocal laser scanning microscope with spectral detection (Leica Microsystems, Inc.) and an HCX PL APO 63 × NA 1.4 oil immersion objective. Alexa Fluor 488 was excited at 488 nm, and emission was detected between 500 to 550 nm. DAPI was excited at 405 nm, and emission was detected between 420 to 470 nm. Imaging was performed in sequential scan mode. Sampling was performed with a pixel size of 40 × 40 nm (x/y). Fluorescence and transmission images were acquired simultaneously. To verify specificity of the procedure, the parental strain not producing HA-tagged protein was treated with the immunolabeling procedure and inspected under the microscope.

### Sterol analysis

Extraction and quantification of *K. phaffii* sterols were performed as previously described by Ott *et al.* (2005) with minor modifications (63). Briefly, cells corresponding to 15 OD_600_ units of exponentially growing cultures (OD_600_ = 3) were harvested by centrifugation at 4000×*g* for 10 min in Pyrex glass tubes. The supernatant was discarded, and the pellet was resuspended in a mixture consisting of 0.6 ml methanol, 0.4 ml pyrogallol (0.5% in methanol), and 0.4 ml KOH (60% w/v in water). Cholesterol (1–2 mg) was added as an internal standard. Samples were incubated at 90 °C for 2 h to facilitate cell lysis and saponification. After cooling to room temperature, sterols were extracted with 1 ml n-heptane using a VXR basic Vibrax shaker (IKA) at 1500 rpm for 10 min. The samples were centrifuged at 500×*g* for 5 min, and the upper (organic) phase was collected. The aqueous phase was subjected to a second extraction with 1 ml n-heptane, and the pooled organic phases were evaporated under a stream of nitrogen. The dried sterol residues were resuspended in 10 μl pyridine, followed by derivatization with 50 μl N,O-bis(trimethylsilyl)trifluoroacetamide (BSTFA) for 20 min at room temperature. Samples were subsequently diluted with 100 μl ethyl acetate prior to gas chromatography–mass spectrometry (GC-MS) analysis. GC-MS was performed using a Shimadzu GC-2010 Plus gas chromatograph equipped with an AOC-20i auto-injector and a GCMS-QP2010 SE mass selective detector. Sterols were separated on a ZB-5MS capillary column (Phenomenex) with the following temperature program: 100 °C to 250 °C at 10 °C/min, followed by 250 °C to 300 °C at 3 °C/min. Helium was used as the carrier gas at a constant flow rate of 0.9 ml/min (linear velocity: 35.3 cm/s). One microliter of sample was injected using a split ratio of 1:20. The detector was operated in electron ionization (EI) mode at 70 eV, in full scan mode (100–550 amu) with a scan rate of 2.56 scans/s.

Sterol compounds were identified based on their characteristic mass fragmentation patterns and their relative retention times compared to the cholesterol internal standard. Measurements were normalized using the internal cholesterol standard and to cell dry weight, determined by filtering yeast cultures through pre-weighed cellulose acetate filters (0.2 μm pore size, Sartorius), followed by drying at 60 °C for 16 h.

### Analysis of *ERG* gene expression

Total RNA was isolated using the SV Total RNA Isolation System (Promega) as previously described by Radkohl *et al.* (25). *K. phaffii* strains were cultivated in MD-his medium for at least 12 h to reach an OD_600_ of 3. Subsequently, 18 OD_600_ units were harvested by centrifugation and stored at −20 °C until further processing. Cell pellets were resuspended in 200 μl resuspension buffer containing 1 M sorbitol, 0.1 M EDTA, and 0.1% (v/v) 2-mercaptoethanol. The suspension was transferred to tubes containing glass beads (0.3–0.5 mm; approximately half the sample volume), and cells were disrupted by eight cycles of 30 s vortexing followed by 30 s incubation on ice. The lysate was centrifuged for 30 s at 1600 rpm, and the supernatant was transferred to a fresh tube. 150 μl of precooled RNA lysis solution were added, and the samples were gently inverted five times. Subsequent RNA purification steps followed the manufacturer’s protocol (SV Total RNA Isolation System, Promega). RNA quality and concentration were assessed by agarose gel electrophoresis and spectrophotometric analysis. Purified RNA samples were stored at −80 °C until further use.

Quantitative reverse transcription PCR (RT-qPCR) was performed using the Luna Universal One-Step RT-qPCR Kit (New England Biolabs) with 200 ng of total RNA as input. Reactions were run on a Rotor-Gene Q thermocycler (Qiagen), following the manufacturer’s protocol. Specific primer pairs for eleven *ERG* genes and two housekeeping genes (*RSC2* and *TAF10*, (64)) used for normalization are listed in Table S6. Each reaction included no-reverse transcriptase and no-template controls to verify the absence of genomic DNA contamination and primer-dimer artifacts. All measurements were performed in technical triplicates and confirmed in two independent experiments. Initial data analysis, including quantification cycle (Cq) determination and melting curve analysis, was carried out using Q-Rex software (Qiagen). Cq values were normalized to the geometric mean of the two housekeeping genes, and fold changes relative to the wild-type strain were calculated using the 2ˆ–ΔΔCq method (65).

### Pull-down assay

#### FLAG-tag immunoprecipitation assay

Cells corresponding to 120 OD_600_ units were harvested by centrifugation at 4 °C and 4000×*g* for 10 min. The pellets were washed with ice-cold water and centrifuged again for 10 min at 4 °C and 3000×*g*. The resulting cell pellet was stored at −20 °C until further use.

The cell pellet was resuspended in 300 μl of 2× TNEG buffer (100 mM Tris-HCl, pH 7.4; 300 mM NaCl; 20% glycerol; 2 mM EDTA, pH 8) supplemented with PMSF (0.1 mM) and cOmplete Mini, EDTA-free protease inhibitor (Roche). Cells were disrupted by the addition of glass beads (0.3–0.5 mm, half the sample volume) and vortexed for 3 min. Lysed cells were centrifuged for 5 min at 1000×*g*. The supernatant was transferred to a new 1.5 ml tube, diluted with 2× TNEG buffer to a final volume of 1 ml, and centrifuged again for 10 min at 1000×*g*.

Anti-FLAG M2 Affinity Gel (35 μl per sample; Millipore) was washed three times with 1× TNEG buffer. The cell lysate was incubated with the washed affinity beads for 1 h at 4 °C on a rotary shaker. After incubation, the beads were washed four times with 1× TNEG buffer. The supernatant was discarded, and beads were mixed with 40 μl of SDS loading buffer (1× Nupage sample buffer, 0.33 M Tris-base, 2% mercaptoethanol) and incubated at 65 °C for 15 min. The eluted samples were then used for immunoblotting.

#### Photoclick assay

Photoclick assays were performed as described previously (35). Briefly, yeast cultures were grown in 500 ml MD-his medium for 15 h at 28 °C and 120 rpm in 3-L baffled flasks until an OD_600_ of three was reached. Cells were harvested by centrifugation (10 min, 4000 g, 4 °C), washed with ice-cold water, and centrifuged again (10 min, 3000 g, 4 °C). Pellets were resuspended in 25 ml TE buffer (20 mM Tris-HCl pH 8, 5 mM EDTA), supplemented with 0.1 mM PMSF and cOmplete Mini protease inhibitor. Cells were lysed using a bead beater (Merckenschlager) for 4 min with CO2 cooling. Lysates were centrifuged (10 min, 3000 g) to remove unlysed cells and beads, followed by ultracentrifugation (45 min, 100,000 g, 4 °C). The resulting membrane pellet was resuspended in 1 ml PBS.

Photoclick assays were performed as described with slight modifications (66). Membrane fractions (175 μl) were incubated with 25 μl Photoclick Cholesterol (960 μM) for 1 h at room temperature. The samples were illuminated with 365 nm UV light for 20 min, then incubated with washed Dde Azide beads (80 μl/sample) in PBS containing 0.5% SDS. To initiate the click reaction, a master mix was prepared (20 μl TCEP, 60 μl TBTA, 20 μl CuSO4) and added to the bead samples, followed by 3 h of incubation on a rotary wheel at room temperature. Beads were washed five times with wash buffer (100 mM Tris-HCl pH 7.4, 1% SDS, 250 mM NaCl, 5 mM EDTA). Elution was achieved with 25 μl 2% hydrazine-dihydrochloride for 2 h at room temperature. Eluted samples were mixed with LDS sample buffer and reducing agent for further immunoblot analysis.

### Structural modeling

The structure of *K. phaffii* Scap protein was attained with the AlphaFold3 Server (36). The ligand for the docking simulations (ergosterol) was retrieved from PubChem (67). A complete and a truncated version of the protein were utilized for the simulation, with the latter containing the central transmembrane helices suspected for sterol interaction (D277-L448). Ligand docking was performed using the SwissDock server *via* AutoDock VINA (68, 69). The search space was defined to encase the entirety of the sterol sensing domain. The results were evaluated by binding affinity and ligand orientation. For the visualization of the protein orientation within the ER membrane, the OPM server was used (PPM 3.0) (38). The respective images were prepared using PyMol (The PyMOL Molecular Graphics System, Version 3.1.1, Schrödinger, LLC).

## Data availability

All data are available in the article itself and its online supplementary materials.

## Supporting information

This article contains supporting information (25, 35, 53, 55, 69, 70, 71).

## Conflict of interest

The authors declare that they have no conflicts of interest with the contents of this article.
