# Supplementary material for: Sterol regulation in Komagataella phaffii: Identification of sterol regulatory element binding proteins and their activation pathway
Source: J Biol Chem. 2025 Aug 8;301(9):110579. doi: 10.1016/j.jbc.2025.110579 (PMC12926077; doi:10.1016/j.jbc.2025.110579)
Supplement: Supplementary Material [file mmc1.docx]

"**Sterol Regulation in *K. phaffii*:
 Identification of Sterol Regulatory Element Binding Proteins and their Activation Pathway**"

Simon Arhar^1^, Melanie Merl^1^, Odysseas Pantelakis^1^, Franziska Gruber^1^, Paula Berzak^1^, Heimo Wolinski^2,3^ and Anita Emmerstorfer-Augustin^1,4,5^*

^1^ Institute of Molecular Biotechnology, Graz University of Technology, NAWI Graz, Graz, Austria

^2^ Institute of Molecular Biosciences, University of Graz, Graz Austria

^3^ BioHealth-Graz, Graz, Austria

^4^ Austrian Centre of Industrial Biotechnology, acib GmbH, Graz, Austria

^5^ BioTechMed-Graz, Graz, Austria

*Corresponding Author Contact:

DI Dr. Anita Emmerstorfer-Augustin

emmerstorfer-augustin@tugraz.at

ORCID ID: 0000-0002-3392-8839


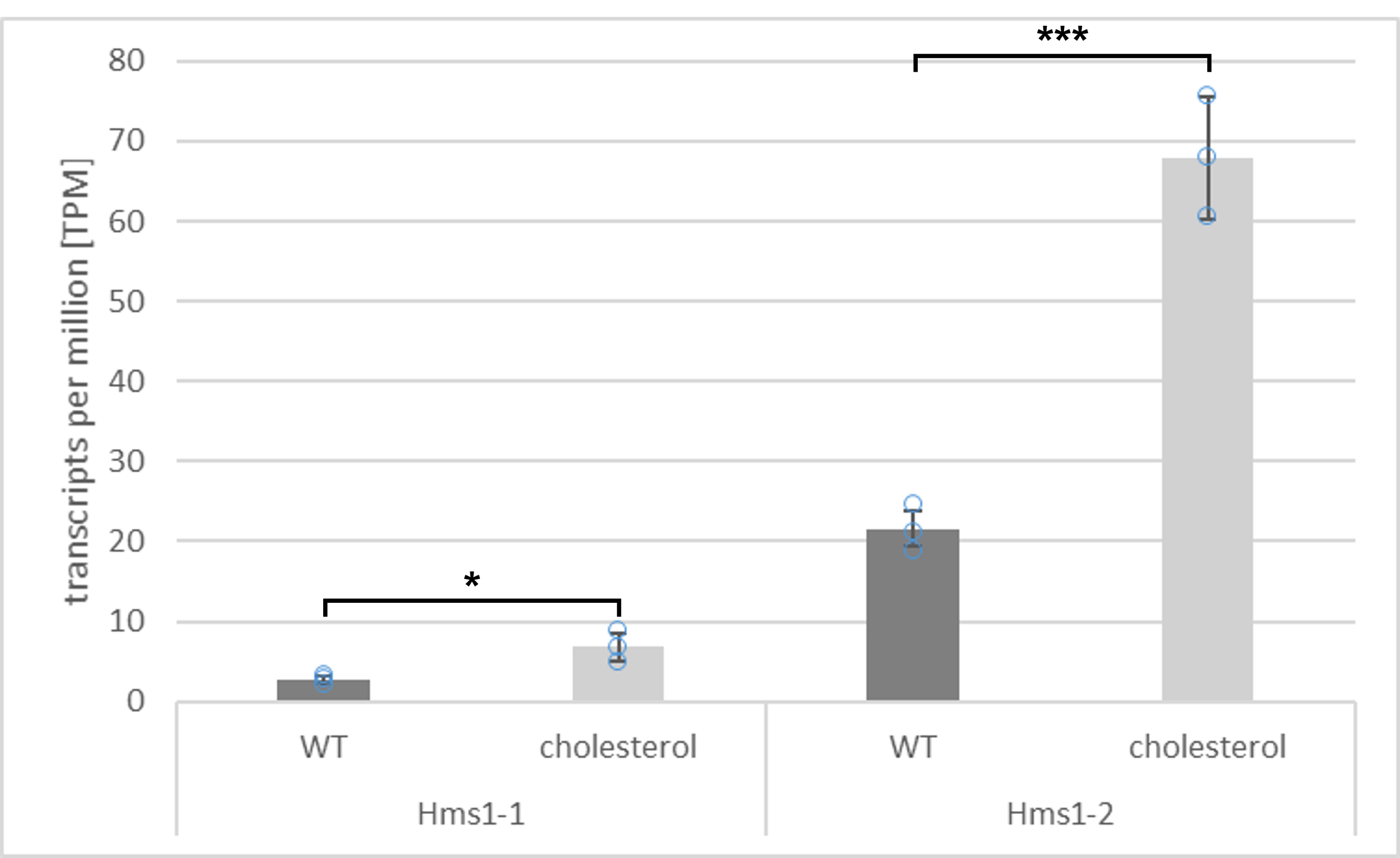


Figure S1:Expression levels of HMS1-1 and HMS1-2 in wild type K. phaffii (CBS4735) and a strain producing cholesterol instead of the native ergosterol (MH458). Transcripts per million from an RNAseq experiment (TPM, fragment counts normalized for gene length) are presented as mean +/− standard deviations from three biological replicates (bar chart). Individual data points are shown as scatter plot. The results from an unpaired two-tailed t-test are indicated by asterisk (*= p<0.05; **= p<0.01; ***=p<0.001).


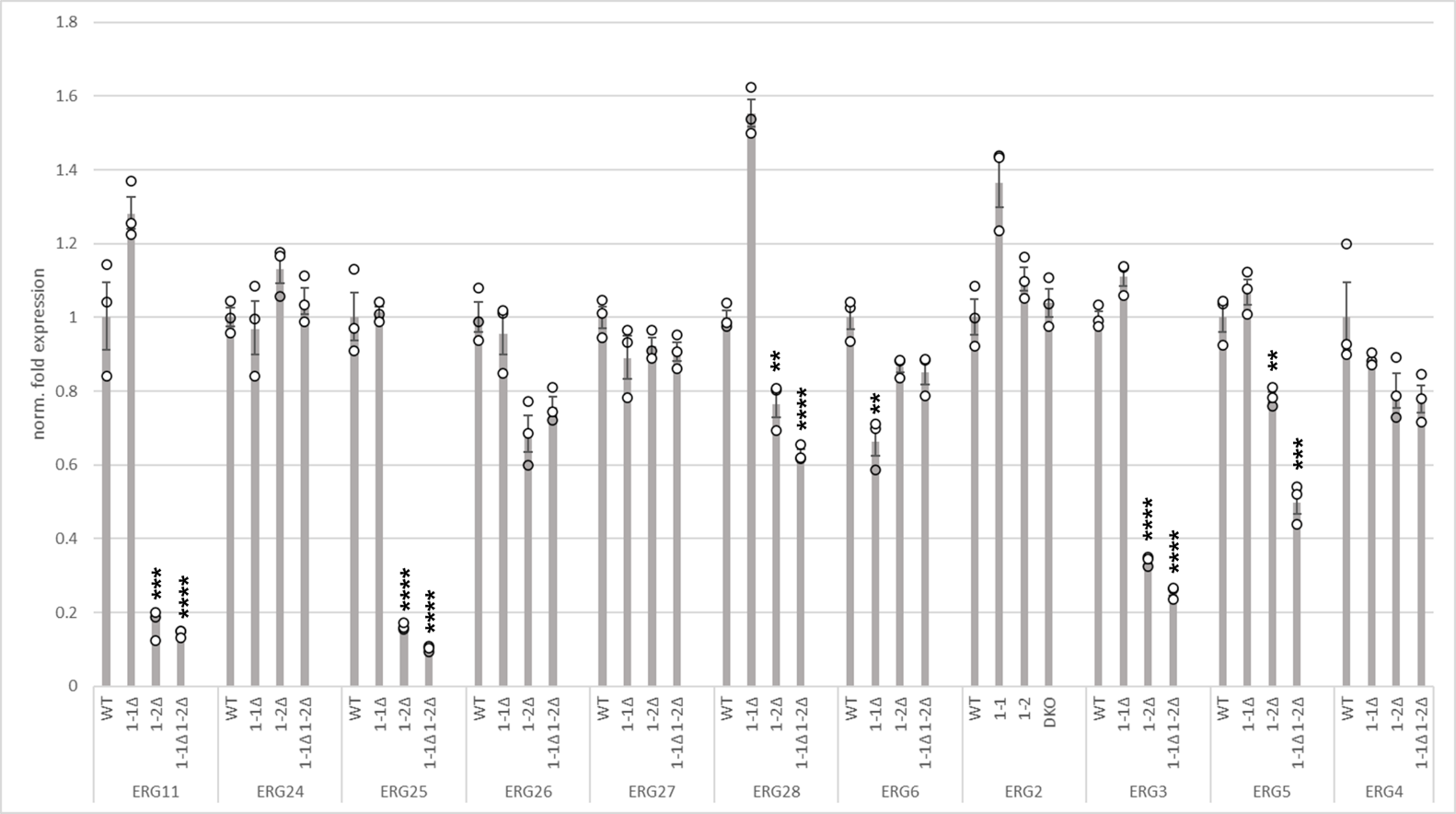


Figure S2: Differential expression of ERG genes in hms1-1Δ (yFG046), hms1-2Δ (yFG051), and hms1-1Δ hms1-2Δ (yFG60) knockout strains. All values are calculated from qRT-PCR results representing biological replicas (n=3). The geometrical mean of 2^(-∆∆Cq) values relative to the wild type are depicted as bar charts with indicated standard errors of the mean. Additionally shown as scattered plot are the individual 2^(-∆∆Cq) values. Results from an unpaired two tailed t-test of the ∆∆Cq values are indicated by asterisk (**= p<0.01; ***=p<0.001; ****=p<0.0001). Data was normalized to two K. phaffii houskeeping genes (RSC2, TAF10). Strains were grown to OD_600_ of 3 in MD-his media.


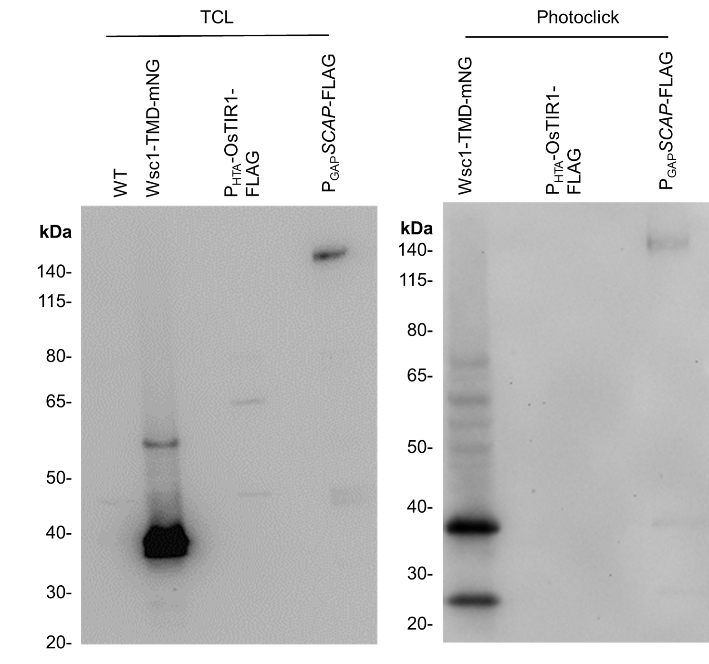


Figure S3: To assess the sterol-binding capability of K. phaffii Scp, we performed a pull-down assay using a photoclickable sterol analog. Scp was C-terminally tagged with FLAG. As a positive control, we used the transmembrane domain of K. phaffii Wsc1 (Wsc1-TMD), known to interact with sterols (1). The soluble and FLAG-tagged Oryza sativa TIR1 (OsTIR1), with no known sterol interactions(2), served as a control to evaluate potential nonspecific interactions with the FLAG epitope. Wild-type K. phaffii was included to assess the specificity of the anti-FLAG antibody. Shown are the untreated total cell lysates (TCL) and the protein fractions obtained after incubation with photoclick sterol and subsequent pull-down. Expected molecular weights: Wsc-TMD-mNG, 35kDa; OstTIR, 64 kDa; Scp, 147 kDa.


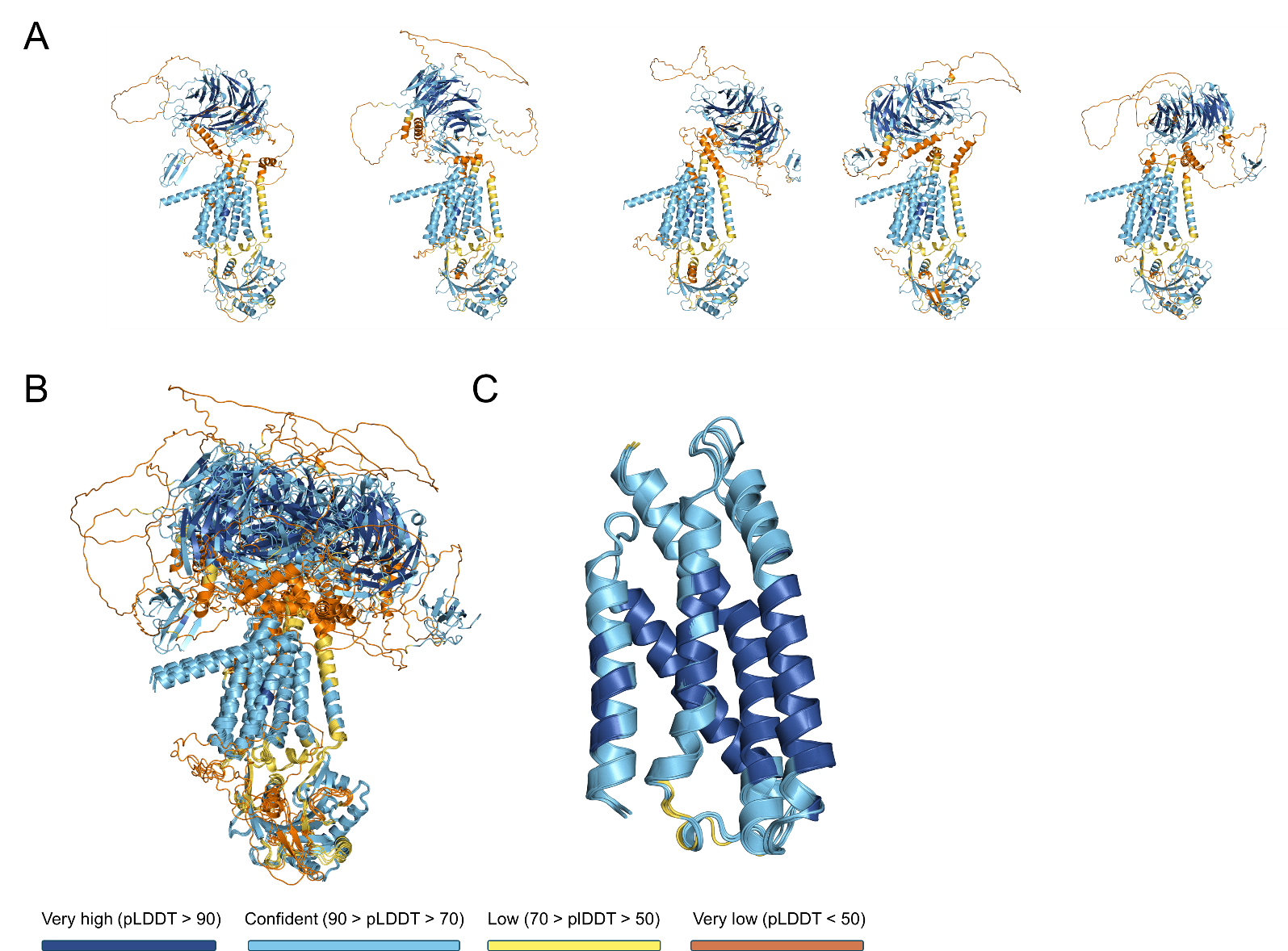


Figure S4: Prediction of K. phaffii Scp protein structure by Alphafold 3. **(A)** The five best predicted models for K. phaffii Scp are shown separately. **(B)** The structural alignment of the best predictions indicates highly similar folds of the transmembrane domain and ER lumen located domain, while the cytosolic WD-domain is differently oriented in each model due to the regions of low confidence, which are handled as flexible loops by the prediction algorithm. **(C)** Especially the fold of sterol sensing domain is conserved between the five predictions. Colors shown within the predicted structures are indicative for the results of the predicted local distance difference test (pLDDT).

Table S1: Results of the 10 lowest energy dockings of ergosterol to the predicted K. phaffii Scp sterol sensing domain using the SwissDock server. Shown are the calculated affinities and the positions of ergosterol in the sterol sensing domain. Affinities of dockings indicating ergosterol in the same hydrophobic pocket are indicated in green.

| **No** | **Calculated affinity (kcal/mol)** | **Docking position** | **No** | **Calculated affinity (kcal/mol)** | **Docking position** |
| --- | --- | --- | --- | --- | --- |
| 1 | -7.650 | 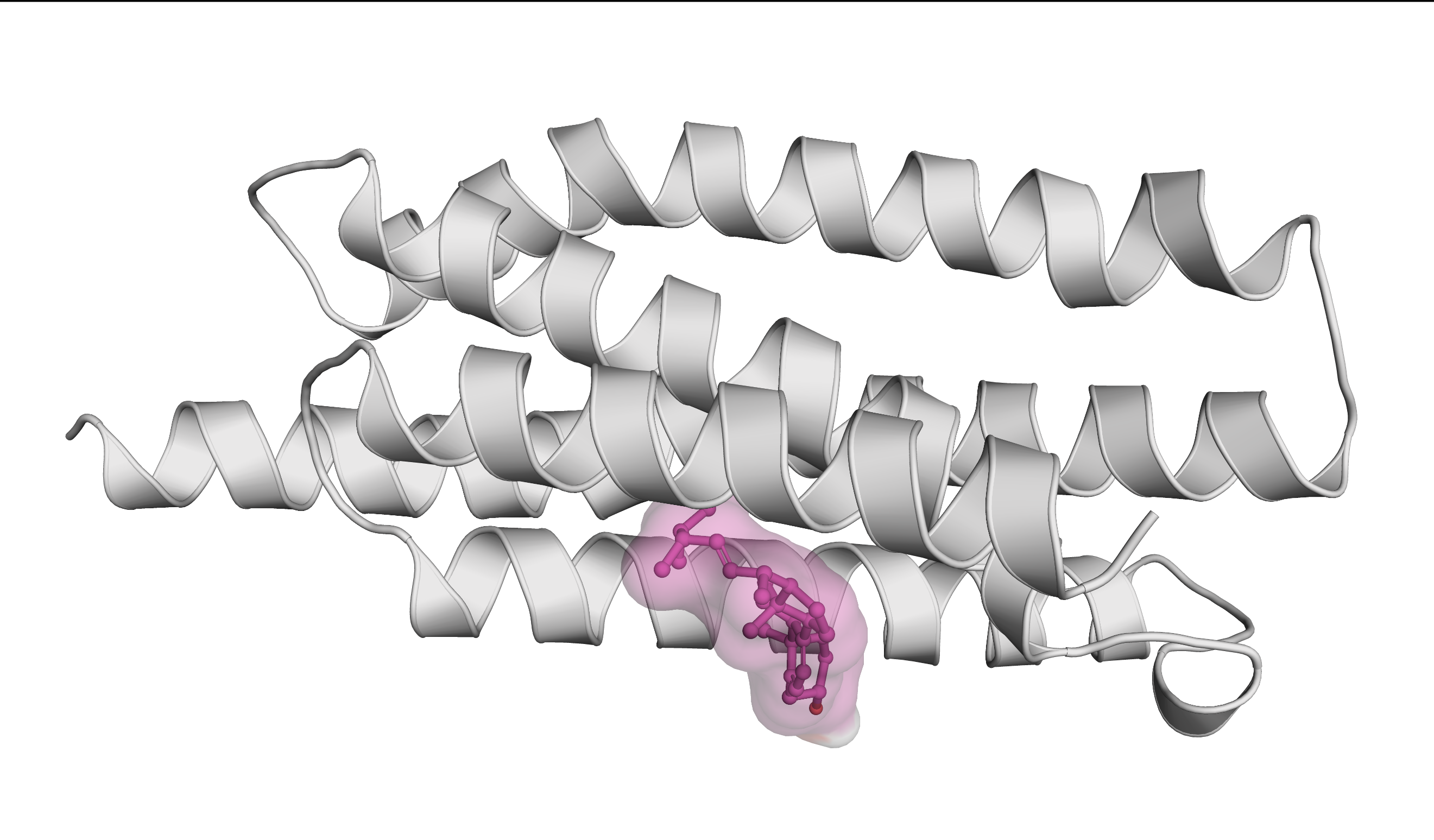 | 6 | -6.848 | 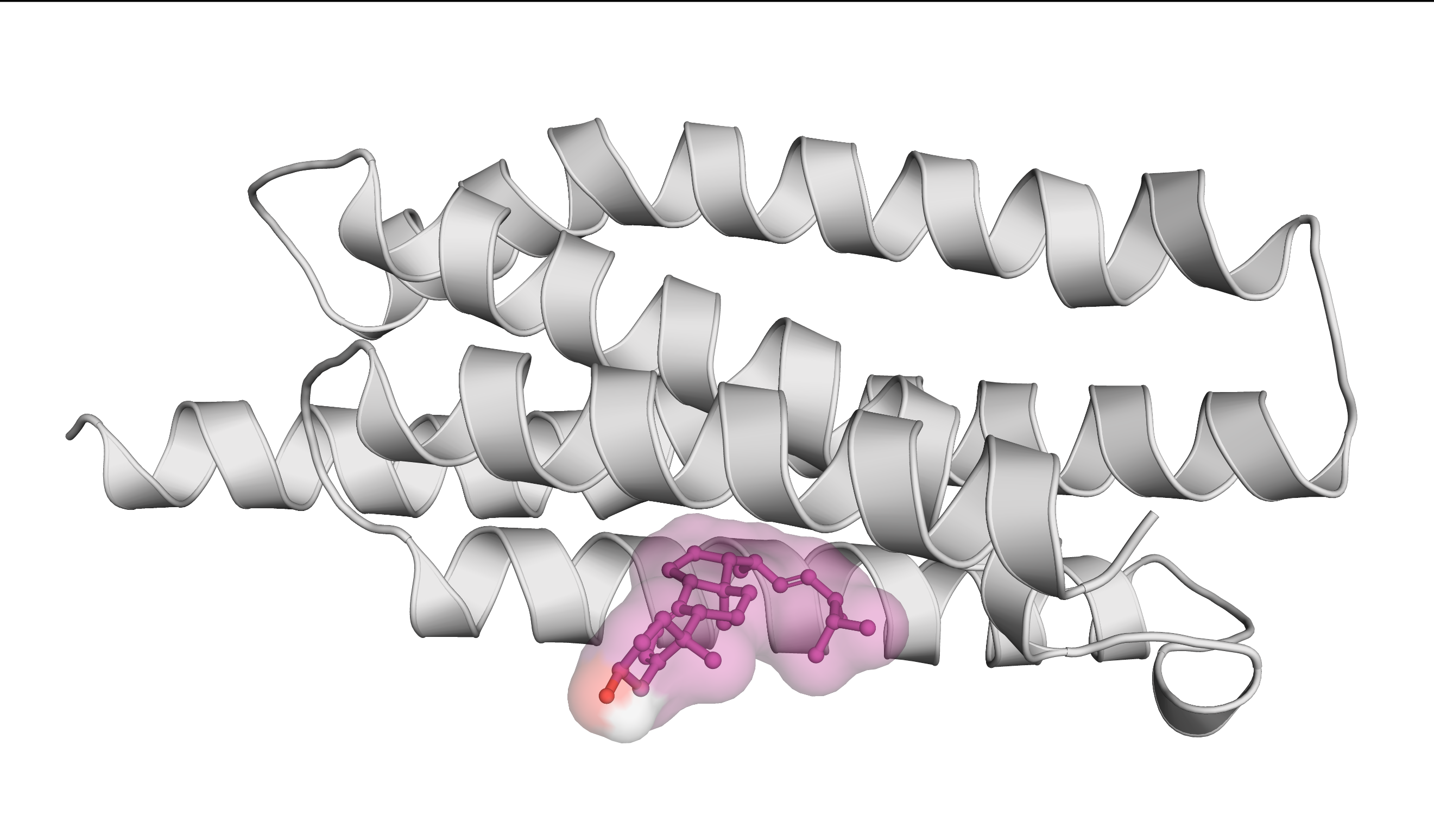 |
| 2 | -7.102 | 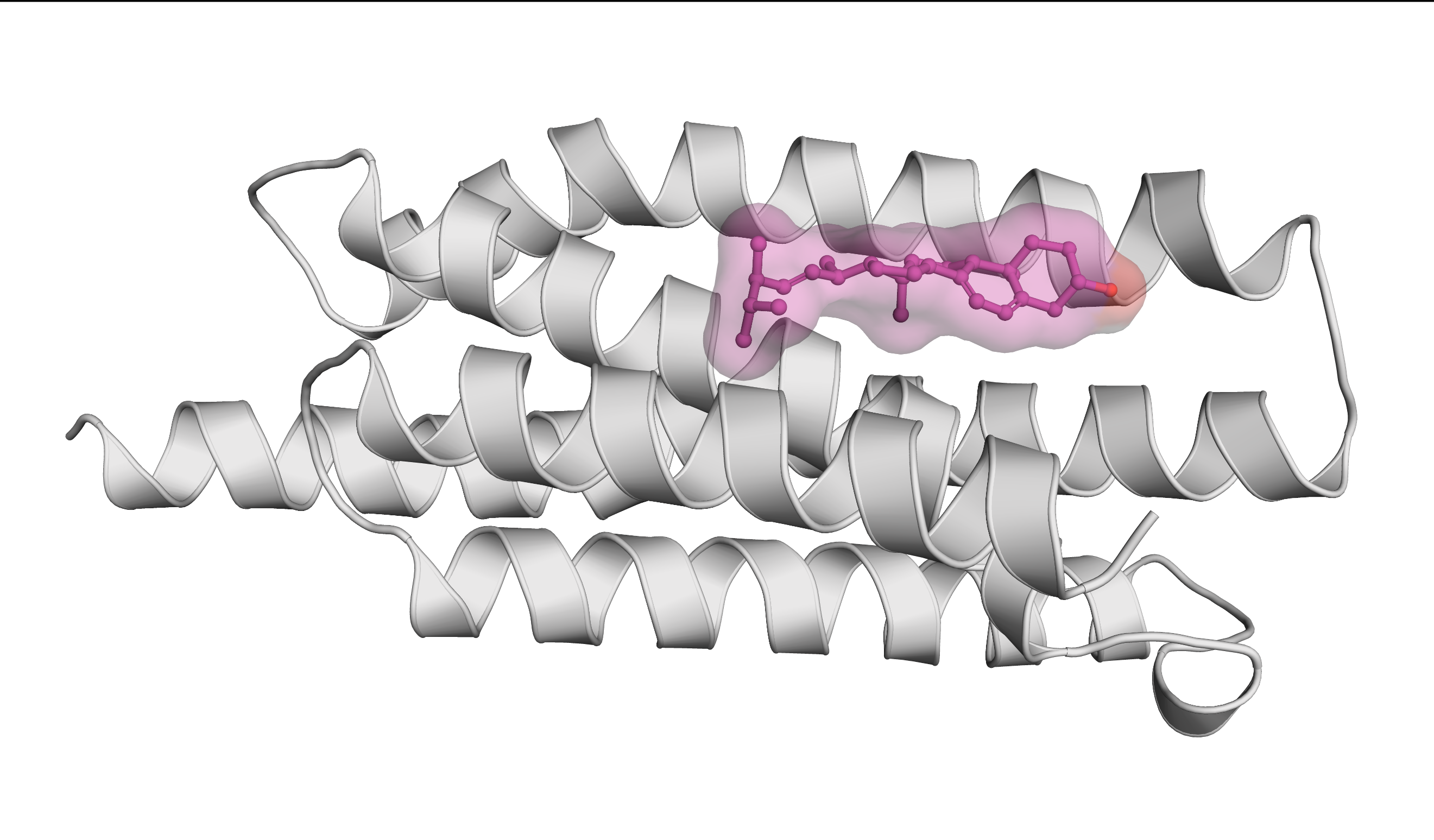 | 7 | -6.751 | 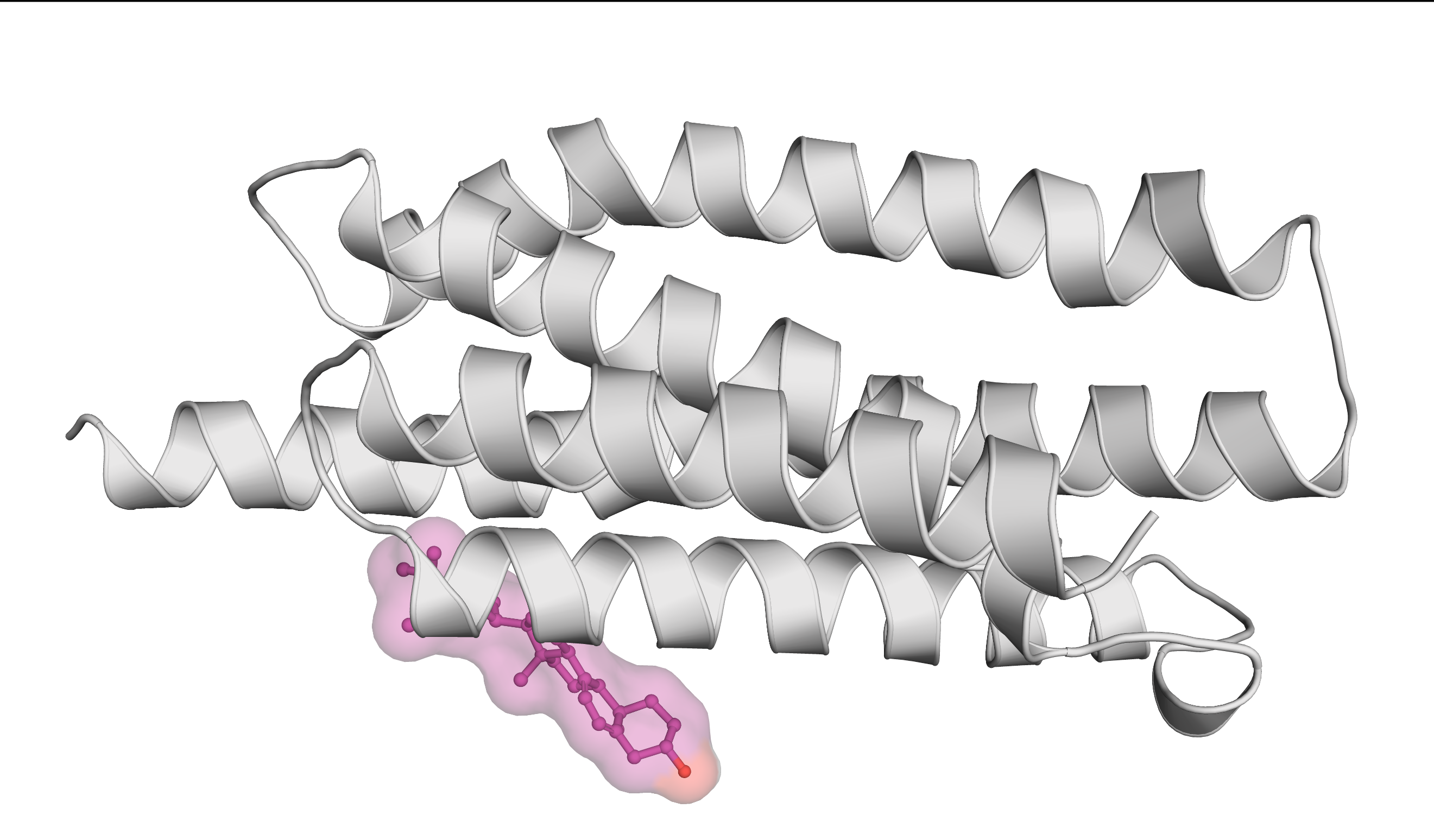 |
| 3 | -7.033 | 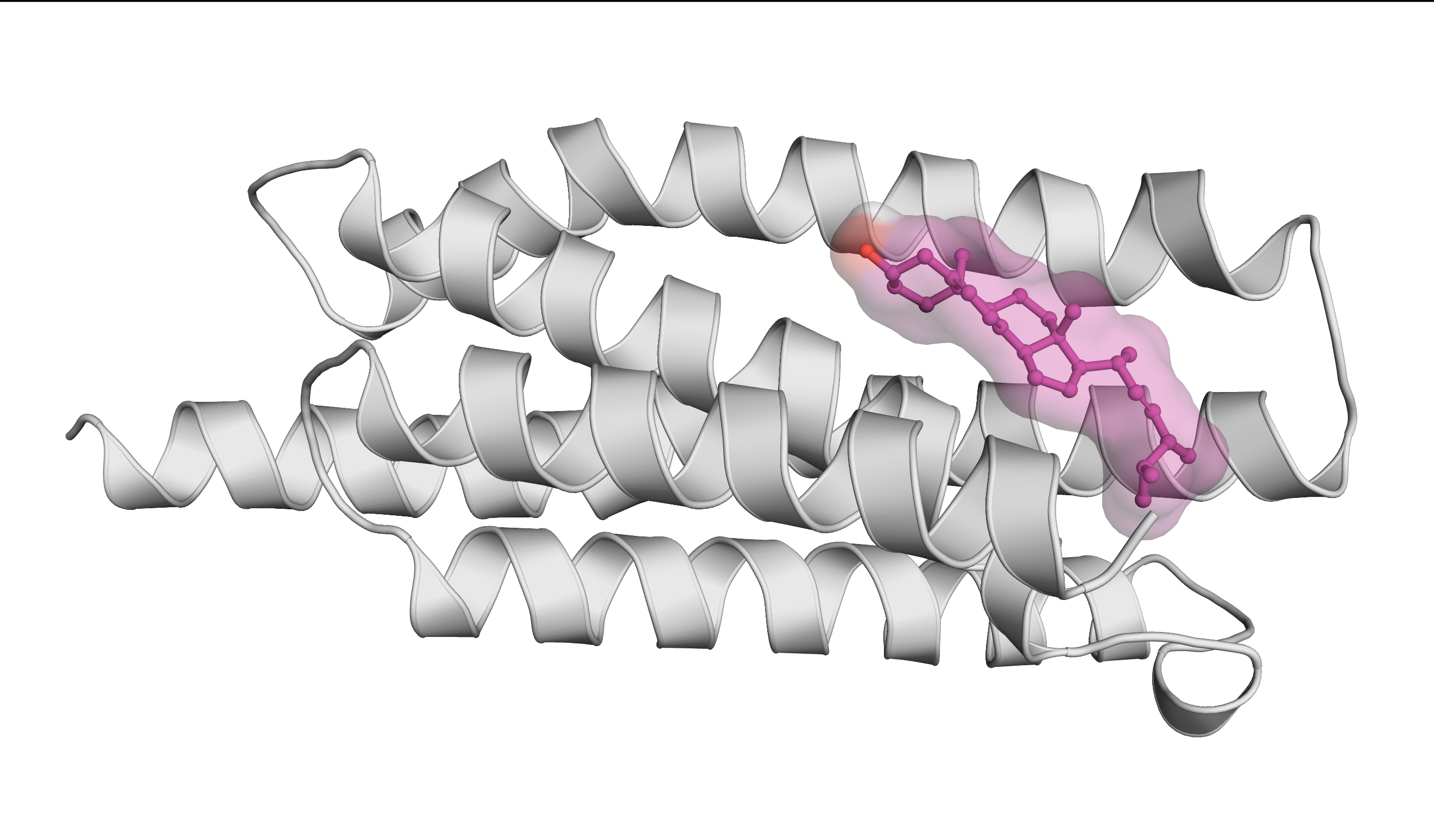 | 8 | -6.549 | 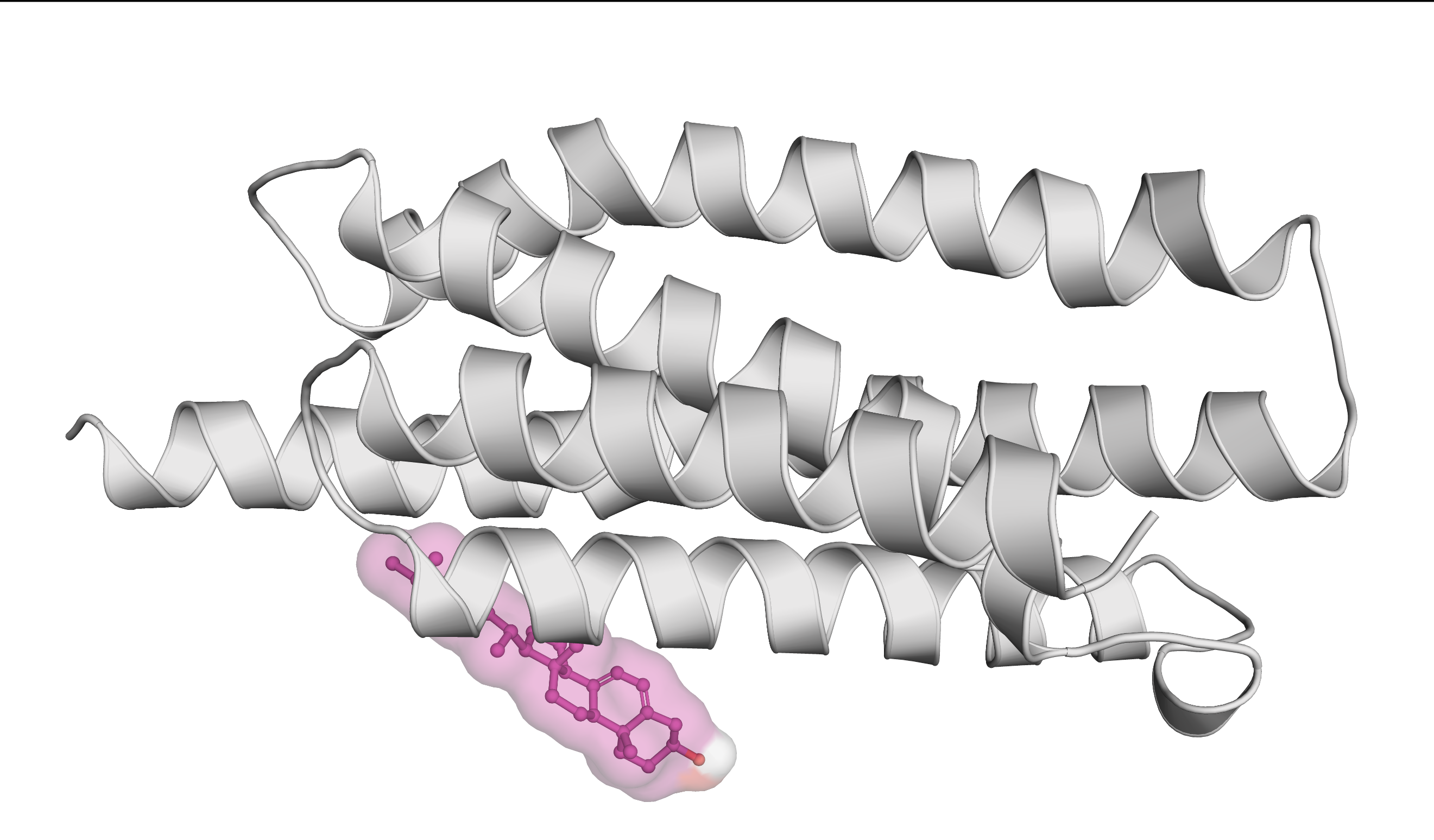 |
| **4** | **-7.013 (shown in pic)** | **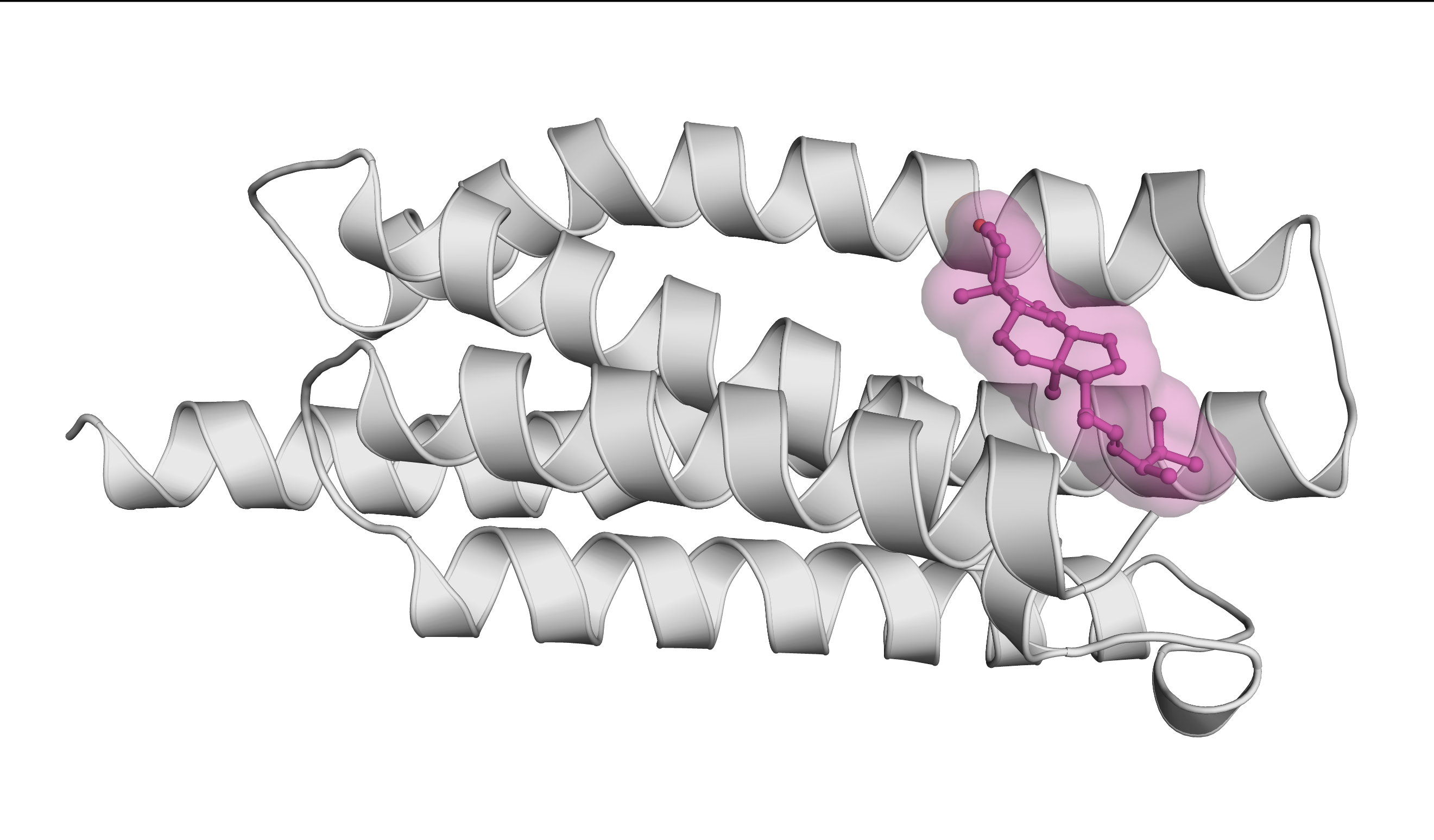** | 9 | -6.455 | 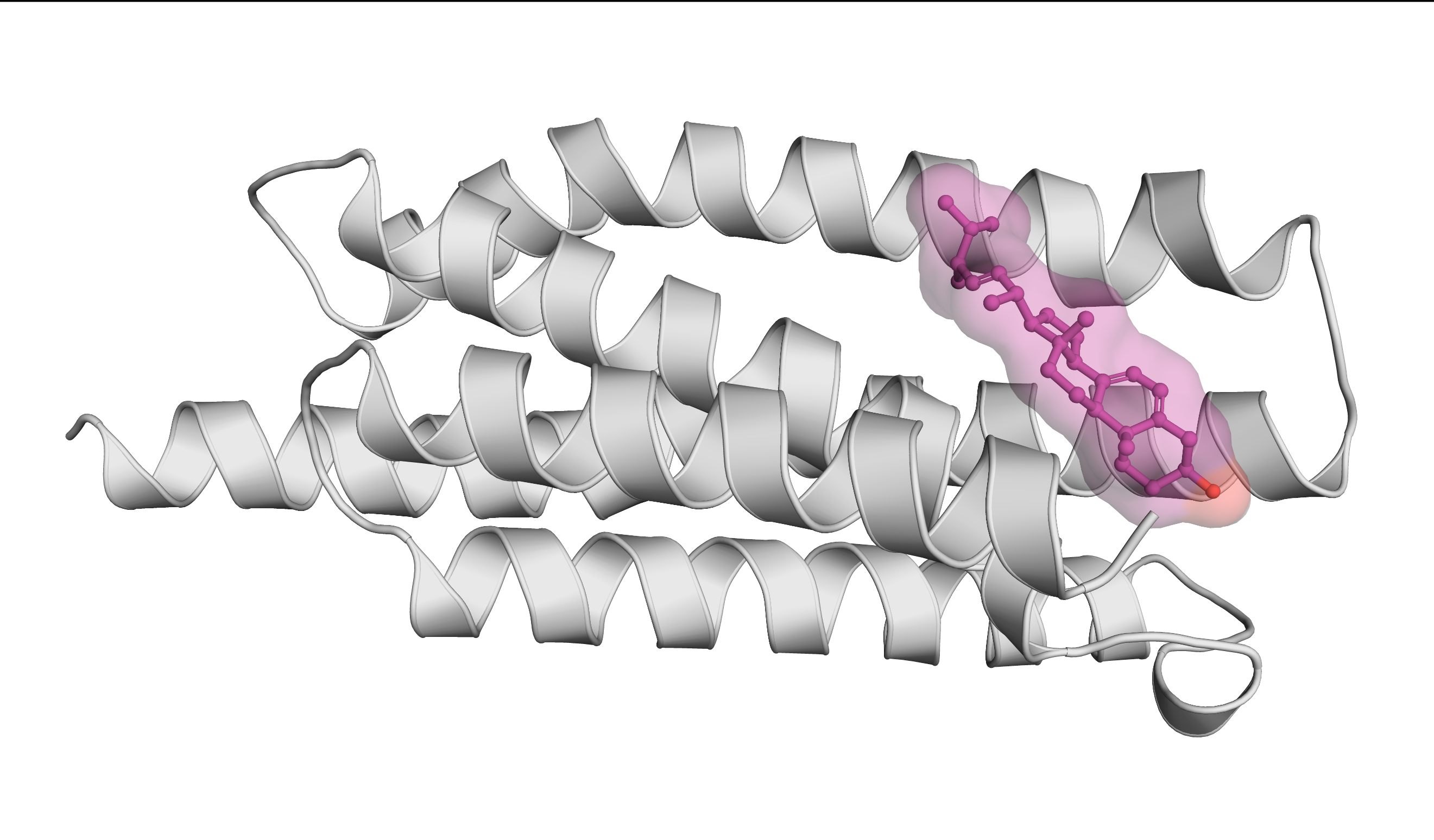 |
| 5 | -6.994 | 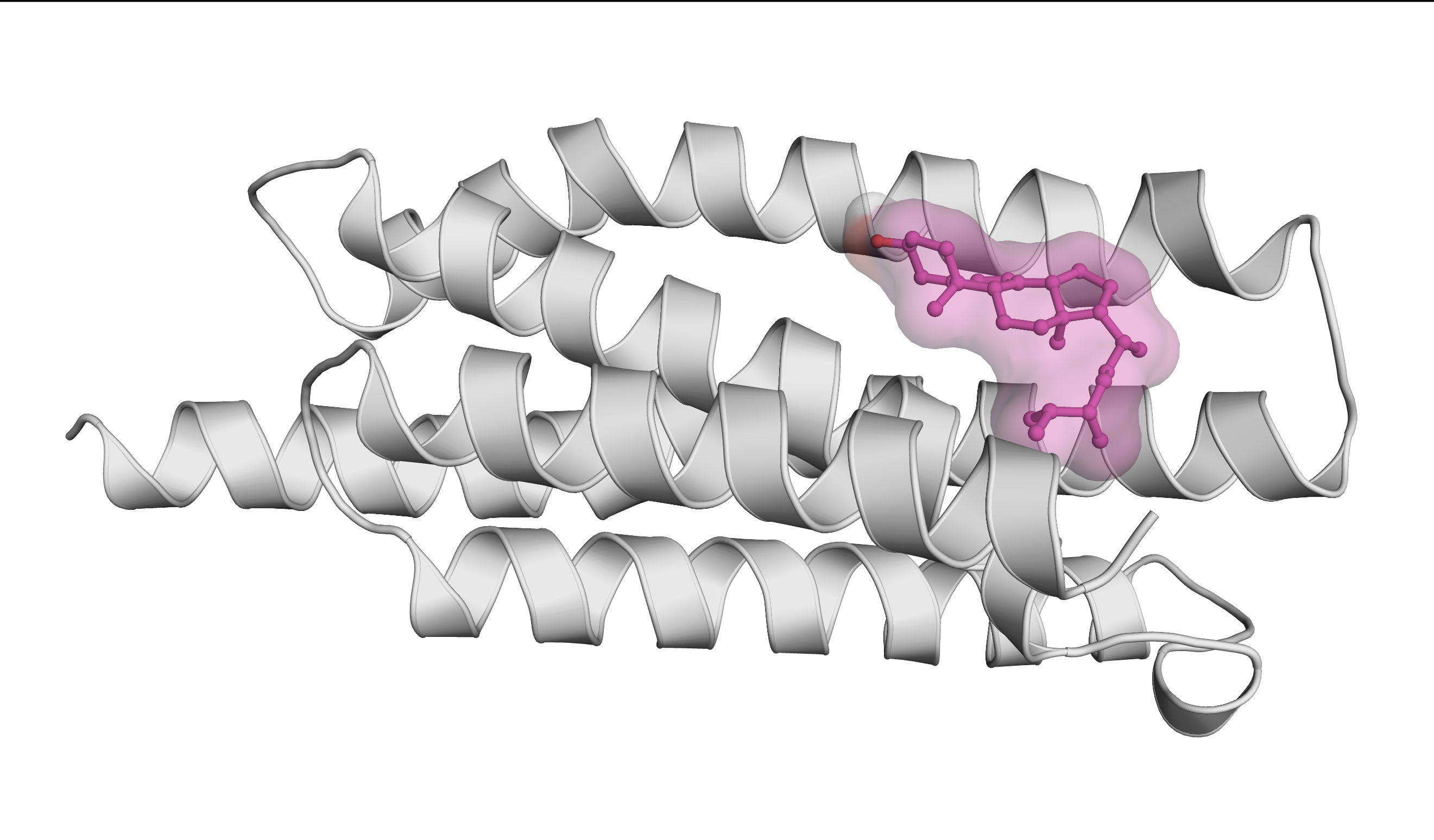 | 10 | -6.443 | 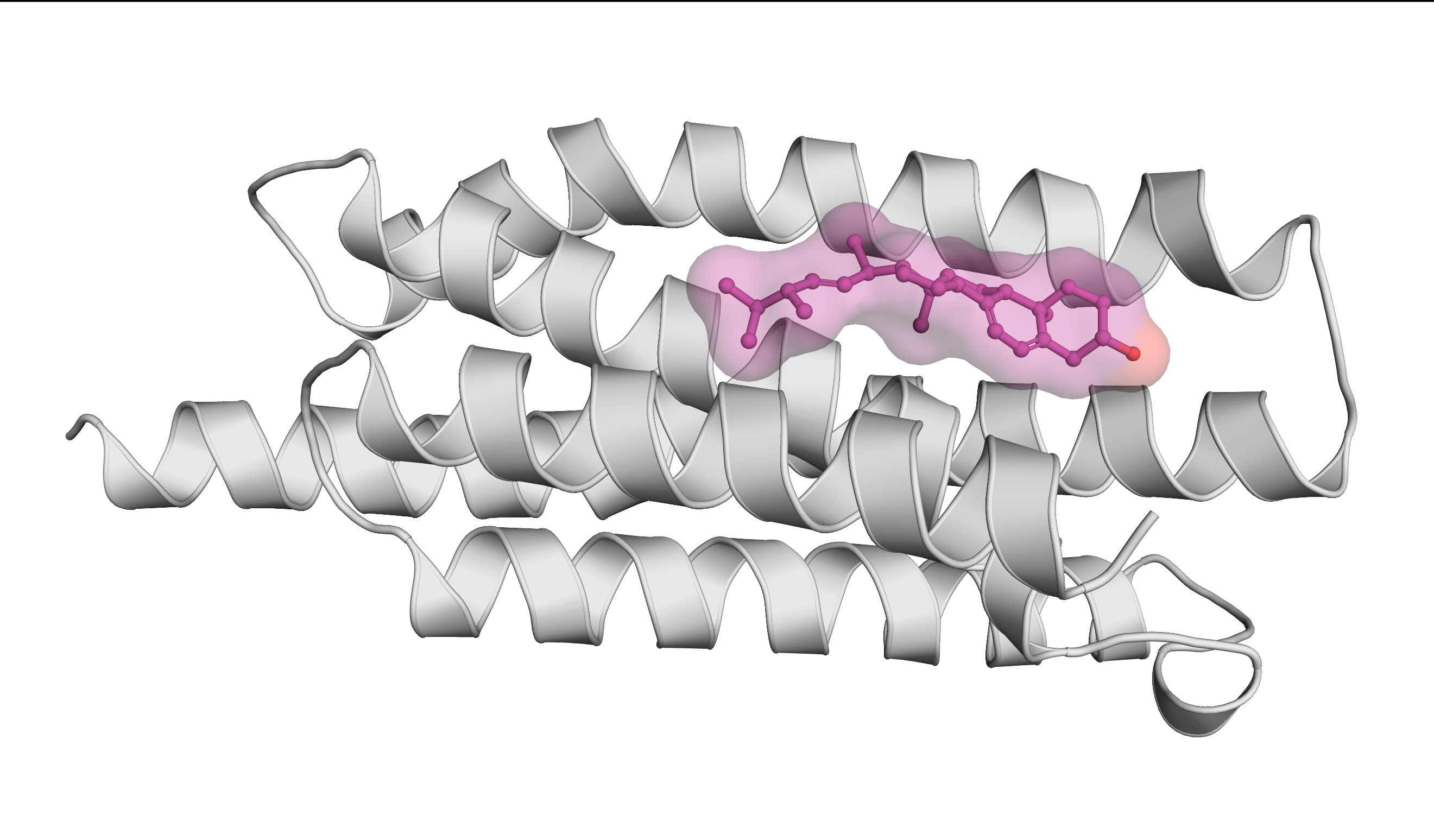 |

Table S2: Plasmids used in this study.

| **Plasmid** | **Description** | **Application** | **Source** |
| --- | --- | --- | --- |
| pPpKC2 | pUC ori, G418^R^, AMP^R^, *P_AOX_*-Flippase | Vector backbone | (3) |
| pPpKC2-Wsc1-AID*-3HA | *WSC1*-Aid, 3xHA, | 3xHA template | (4) |
| pPpKC2-his4-TEF1prom-RHO1 | pUC ori, G418^R^, AMP^R^, *P_AOX_*-Flippase, 5’HR-*HIS4*, 3’HR-*HIS4*, *P_KpTef2_*-*RHO1* | Vector backbone | (4) |
| pPpKC2-his4del-TEF1-3HA-Hms1-1-V5 | pPpKC2-his4, *P_KpTEF1_*-3xHA-*HMS1-*1-V5 | *HMS1-1* overexpression | This study |
| pPpKC2-his4del-TEF1-3HA-Hms1-2-V5 | pPpKC2-his4, *P_KpTEF1_*-3xHA-*HMS1-2*-V5 | *HMS1-2* overexpression | This study |
| PpPKC2_UPC2-AID-Strep | pPpKC2-his4-, 5’HR-*UPC2*, 5’HR-*UPC2*, AID-STREP-tag | AID-Strep tagging of *UPC2* | This study |
| A179-Hyg | pUC, Hyg^R^, PARS1, *HsCas9*, sgRNA | CRISPR/Cas9 | (4) |
| A179-Hyg-Hms1-1ko | A179-Hyg, *HMS1-1* protospacer | *HMS1-1* deletion | This study |
| A179-Hyg-Hms1-2ko | A179-Hyg, *HMS1-2* protospacer | *HMS1-2* deletion | This study |
| A179-Hyg-dual_his4-del | A179-Hyg, *HIS4* protospacer | *HIS4* deletion | This study |
| A179Hyg_End_Upc2 | A179-Hyg, *UPC2* protospacer-stop | *UPC2* AID-tagging | This study |
| A179Hyg_Start_Upc2 | A179-Hyg, *UPC2* protospacer-start | *UPC2* deletion | (5) |
| A179Hyg-startSCP | A179-Hyg, *SCP* protospacer | *SCP* deletion | This study |
| A179Hyg-startDSC1 | A179-Hyg, *DSC1* protospacer | *DSC1* deletion | This study |
| pPpT4GAP_S | pUC ori, Zeo^R^, *P_GAP_* | Vector backbone | (6) |
| pPpT4GAP-SCP-Flag | pPpT4GAP_S, *SCP*-FLAG | *SCP* overexpression | This study |
| pPp T4 Pgut1-HMS1-1 | pPpT4, *P_KpGUT1_-HMS1-1* | production of Hms1-1 | This study |
| pPp T4 Pgut1-1^N-268^ | pPpT4, *P_KpGUT1_-HMS1-1^N-268^* | production of truncated Hms1-1 (amino terminal 268 AAs) | This study |
| pPp T4 Pgut1-1^N-298^ | pPpT4, *P_KpGUT1_-HMS1-1^N-298^* | production of truncated Hms1-1 (amino terminal 298 AAs) | This study |
| pPp T4 Pgut1-1^N-353^ | pPpT4, *P_KpGUT1_-HMS1-1^N-353^* | production of truncated Hms1-1 (amino terminal 353 AAs) | This study |
| pPp T4 Pgut1-1^N-400^ | pPpT4, *P_KpGUT1_-HMS1-1^N-400^* | production of truncated Hms1-1 (amino terminal 400 AAs) | This study |
| pPp T4 Pgut1-HMS1-2 | pPpT4, *P_KpGUT1_-HMS1-2* | production of Hms1-2 | This study |
| pPp T4 Pgut1-2^N265^ | pPpT4, *P_KpGUT1_-HMS1-2^N265^* | production of truncated Hms1-2 (amino terminal 265 AAs) | This study |
| pPp T4 Pgut1-2^N314^ | pPpT4, *P_KpGUT1_-HMS1-2^N314^* | production of truncated Hms1-2 (amino terminal 314 AAs) | This study |
| pPp T4 Pgut1-2^N344^ | pPpT4, *P_KpGUT1_-HMS1-2^N344^* | production of truncated Hms1-2 (amino terminal 344 AAs) | This study |
| pPp T4 Pgut1-1^N298/K110R^ | pPpT4, *P_KpGUT1_-HMS1-1^N298_/^*^K110R^ | production of truncated Hms1-1 (amino terminal 298 AAs) with an lysin to arginine mutation at amino acid 110 | This study |
| pPp T4 Pgut1-1^N298/K191R^ | pPpT4, *P_KpGUT1_-HMS1-1^N298/^*^K191R^ | production of truncated Hms1-1 (amino terminal 298 AAs) with an lysin to arginine mutation at amino acid 191 | This study |
| pPp T4 Pgut1-1^N298/K197R^ | pPpT4, *P_KpGUT1_-HMS1-1^N298/^*^K197R^ | production of truncated Hms1-1 (amino terminal 298 AAs) with an lysin to arginine mutation at amino acid 197 | This study |

Table S3: Primers used during strain construction. Underline indicates protospacer and hammer head sequences.

| **Primer** | **Sequence** | **Application** |
| --- | --- | --- |
| CRISPR-R1 | gggcatcacaatcatggagc | Cloning A179-Hyg plasmids |
| CRISPR-F2 | cctcgagaaagtcgatgggg | Cloning A179-Hyg plasmids |
| rev_AOX_TT | aggagtgggaaataccaag | Sequencing A179-Hyg/pPpT4 plasmids |
| 1-1del_f1 | acgaaacgagtaagctcgtcacgcaccagtacgatgaacggttttagagctagaaatagc | Cloning A179-Hyg-Hms1-1ko |
| 1-1del_r1 | gagcttactcgtttcgtcctcacggactcatcagacgcactttgatttgtttaggtaact | Cloning A179-Hyg-Hms1-1ko |
| pCONT1-1_fw | gtgcctgtttcactgaacgactc | Verify HMS1-1 deletion and tagging |
| pCONT1-1_rev | gctgatcagtcacacttaatcaacag | Verify HMS1-1 deletion and tagging |
| 1-2del_f1 | acgaaacgagtaagctcgtctgaagactacagctctttgcgttttagagctagaaatagc | Cloning A179-Hyg-Hms1-2ko |
| 1-2del_r1 | gagcttactcgtttcgtcctcacggactcatcagtgaagatttgatttgtttaggtaact | Cloning A179-Hyg-Hms1-2ko |
| fw(check_hms1-2) | ctaatggtttcgcgcctcgc | Verify HMS1-2 deletion and tagging |
| REV(check-hms1-2) | gactccctgcatctgtttgg | Verify HMS1-2 deletion and tagging |
| Upcdel-end f1 | acgaaacgagtaagctcgtcgtatatatgatgagtaacgagttttagagctagaaatagc | Cloning A179Hyg_End_Upc2 |
| Upcdel-end r1 | gagcttactcgtttcgtcctcacggactcatcaggtatattttgatttgtttaggtaact | Cloning A179Hyg_End_Upc2 |
| UPC2_fw_seq_start | atggctaacctaaagatccc | Verification of UPC2 knockout and AID-tagging |
| rev_check_UPC2 | atagatacagtccaagc | Verification of UPC2 knockout |
| His4 del_f1 | acgaaacgagtaagctcgtcttatcagtgagtcagtcatcgttttagagctagaaatagc | Cloning A179-Hyg-dual_his4-del |
| His4 del_r1 | gagcttactcgtttcgtcctcacggactcatcagttatcatttgatttgtttaggtaact | Cloning A179-Hyg-dual_his4-del |
| fw_F1_DSC1 | acgaaacgagtaagctcgtcctattactaggtccaaatgggttttagagctagaaatagc | Cloning A179Hyg-startDSC1 |
| rev_F2_DSC1 | gagcttactcgtttcgtcctcacggactcatcagctattatttgatttgtttaggtaact | Cloning A179Hyg-startDSC1 |
| fw_DSC1_up | gaaacctgtgtataccagc | Verify DSC1 deletion |
| rev_DSC1 | catgctcacagttatccg | Verify DSC1 deletion |
| rev_DSC1_seq | tgccctcttccgcactcc | Verify DSC1 deletion |
| fw_SCP_F1 | acgaaacgagtaagctcgtcttgttggaataatacacagcgttttagagctagaaatagc | Cloning A179Hyg-startSCP |
| rev_SCP_F2 | gacgagcttactcgtttcgtcctcacggactcatcagttgttgtttgatttgtttaggtaact | Cloning A179Hyg-startSCP |
| fw_SCP_up | gttagatggtcccttccg | Verify SCP deletion |
| rev_SCP | tcttcaaaatgcccttttcg | Verify SCP deletion |
| fw_SCP_seq | gtgtgtaagtttacctccg | Verify SCP deletion |
| UPC2_F1_fw | ttaagtgagaccttcgtttgtgcggatccatttaaatacagcagctggtatatctgatgc | Cloning PpPKC2_UPC2-AID-Strep |
| UPC2_F2_rev | aaccagatccactacctccggatccagaaccctcatcatatatactactgtgcatgatat | Cloning PpPKC2_UPC2-AID-Strep |
| UPC2_F3_fw | ggttctggatccggaggtagtggatctggttccggaggtagtcgtacgctgcaggtcgac | Cloning PpPKC2_UPC2-AID-Strep |
| UPC2_F4_rev | cctttttattattattgaactaaattaacactcgttatgggtatctagaagcgtaatctg | Cloning PpPKC2_UPC2-AID-Strep |
| UPC2_F5_fw | ccagattacgcttctagatacccataacgagtgttaatttagttcaataataataaaaag | Cloning PpPKC2_UPC2-AID-Strep |
| UPC2_F6_rev | aggcgtatcacgaggccctttcgtcggtaccatttaaatggaggtccgaaaatagactgg | Cloning PpPKC2_UPC2-AID-Strep |
| UPC2_seq_fw | attgcacaatgagcgatac | Sequencing PpPKC2_UPC2-AID-Strep, verification of integration |
| rev_UPC2_check | gccccaagaggaggc | Sequencing PpPKC2_UPC2-AID-Strep, verification of integration |
| F1(HA_1-1_V5) | catacattttagttattcgccaacttaattaaatgagcggttctggttccgg | Cloning pPpKC2-his4del-TEF2-3HA-Hms1-1-V5 |
| R1(HA_1-1_V5) | agcgtaatctggaacgtcgt | Cloning pPpKC2-his4del-TEF2-3HA-Hms1-1-V5 |
| F2(HA_1-1_V5) | ggatcctatccatacgacgttccagattacgctatgaacgaggattataactacgg | Cloning pPpKC2-his4del-TEF2-3HA-Hms1-1-V5 |
| R2(HA_1-1_V5) | cagtttattgtatcattaacaaactcagtatactgcagcatatggcggccgctcacgtagaatcgagaccgaggagagggttagggataggcttaccgttgatgtatccattcagcttc | Cloning pPpKC2-his4del-TEF2-3HA-Hms1-1-V5 |
| F2(HA_1-2_V5) | gatcctatccatacgacgttccagattacgctatggttaatgtgaagtttgaagac | Cloning pPpKC2-his4del-TEF2-3HA-Hms1-1-V5 |
| R2(HA_1-2_V5) | tttattgtatcattaacaaactcagtatactgcagcatatggcggccgctcacgtagaatcgagaccgaggagagggttagggataggcttaccggcatgatctagaaggtttatcc | Cloning pPpKC2-his4del-TEF2-3HA-Hms1-1-V5 |
| Fw(seq_TEFprom) | cgcactcgtacatgattggctg | Sequencing pPpKC2-his4del-TEF1-3HA-Hms-V5 |
| Fw(seq_ARG4tt) | gacagtcagttagtagatatttatac | Sequencing pPpKC2-his4del-TEF1-3HA-Hms-V5 |
| Rv(seq_Hms1-2) | tctttccatatccaccacggg | Sequencing pPpKC2-his4del-TEF1-3HA-Hms1-2-V5 |
| Rv(seq_Hms1-1) | gaagctggagtcaacccttcc | Sequencing pPpKC2-his4del-TEF1-3HA-Hms1-1-V5 |
| pCont-His4_f1 | acctcagccagatcaaagtcac | Verification of pPpKC2-his4del-TEF1-3HA-Hms-V5 insertion |
| pCont-His4_r1 | cgttgcatgttacttgtctccag | Verification of pPpKC2-his4del-TEF1-3HA-Hms-V5 insertion |
| F1(3HA-Hms1-1) | cgcaagcttgttagttcagtttgtttacgcaccagtacg atgagcggttctggttccggg | Overlap extension PCR for endogenous tagging of *HMS1-1* |
| R1(mNG-Hms1-1) | actccgtatcggggtaaccgtagttataatcttcgtt catagcgtaatctggaacgtcgt | Overlap extension PCR for endogenous tagging of *HMS1-1* |
| F1(3HA-Hms1-2) | tctcgtaaacttcttcccttaagaaaattatttcacaaa atgagcggttctggttccggg | Overlap extension PCR for endogenous tagging of *HMS1-2* |
| F1(3HA-Hms1-2) | aagcaaagagctgtagtcttcaaacttcacattaac catagcgtaatctggaacgtcgta | Overlap extension PCR for endogenous tagging of *HMS1-2* |
| SCP-Flag_fw | caattgaacaactatcaaaacacagaattccatgaaagaaattatacatttgatacagac | Cloning pPpT4GAP-SCP-Flag |
| SCP-Flag_rev | catcctcttgagcggccgcttacttgtcatcgtcatctttataatcgtccaaagacgaaacagagt | Cloning pPpT4GAP-SCP-Flag |
| fw_seq_SCP_C-terminus | ggaacaaattctgccc | Sequencing pPpT4GAP-SCP-Flag |
| fw_seq_pGAP | gcatgtcatgagattatt | Sequencing and verification of insertion pPpT4GAP-SCP-Flag |
| pCR-tHMS-Pgut1_f1 | gaacactgaaaaatacacagttattattcatttaaatataccgaaaggtaaacaacttcg | Cloning of pPp T4 Pgut1-HMS plasmids |
| pCR-Pgut1_r1 | tatagtagatatatctgtggtatagtgtgaaaaag | Cloning of pPp T4 Pgut1-HMS plasmids |
| pCR-tHMS_f1 | cttctactttttcacactataccacagatatatctactata atgagcggttctggttccg | Cloning of pPp T4 Pgut1-HMS plasmids |
| pCR-1-1N-bHLH_r1 | tcaggcaaatggcattctgacatcctcttgagctcacaaatgcttgatatactcggtggc | Cloning of pPp T4 Pgut1-1^N-268^ |
| pCR-1-1N-TMD_r1 | aatggcattctgacatcctcttgagctcattttgcattctgcaatattgggaatacatg | Cloning of pPp T4 Pgut1-1^N-400^ |
| pCR-1-1N-Sec_r1 | caggcaaatggcattctgacatcctcttgagctcaagagttagagggaggataactggag | Cloning of pPp T4 Pgut1-1^N-353^ |
| pCR-1-1N-RxxL_r1 | ctcaggcaaatggcattctgacatcctcttgagctcatagaaatcttcggttgagctgcc | Cloning of pPp T4 Pgut1-1^N-298^ |
| pCR-1-2N-bHLH_r1 | caggcaaatggcattctgacatcctcttgagctcacaagtgtttgatgtattcaacagac | Cloning of pPp T4 Pgut1-2^N-265^ |
| pCR-1-2N-GL_r1 | tctctcaggcaaatggcattctgacatcctcttgagctca cgtggaaccaccagctaagc | Cloning of pPp T4 Pgut1-2^N-314^ |
| pCR-1-2N-TMD_r1 | atctctcaggcaaatggcattctgacatcctcttgagctca tggtttcaacgccagcgtg | Cloning of pPp T4 Pgut1-2^N-344^ |
| pCR-T4gut-1-2_r1 | aggcaaatggcattctgacatcctcttgagctcaggcatgatctagaaggtttatcctac | Cloning pPp T4 Pgut1-HMS1-2 |
| pCR-T4gut-1-1_r1 | ggcaaatggcattctgacatcctcttgagctcagttgatgtatccattcagcttcagatc | Cloning pPp T4 Pgut1-HMS1-1 |
| pSeq-pPpT4_f1 | gcctacaatgatgacatttggatttgg | Sequencing pPp T4 Pgut1-HMS |
| pSeq-Pgut1_f1 | tgctgttagctcacgcactccg | Sequencing pPp T4 Pgut1-HMS |
| pSeq-1-2fu_f1 | ttcccgtggtggatatggaaa g | Sequencing pPp T4 Pgut1-HMS |
| pSeq-1-1fu_r1 | atttcaggggtagtggcaatcaac | Sequencing pPp T4 Pgut1-HMS |
| pCont-T4gut_f1 | ttttccttaaacaccctccaaacac | Verification of T4 Pgut1-HMS insertion |
| pCont-T4gut_r1 | cacaaacgaaggtctcacttaatcttc | Verification of T4 Pgut1-HMS insertion |
| pCR-1-1_K110R_r1 | acttcttgtctcacaggaagatacatttggc | Cloning of pPp T4 Pgut1-1^N298/K110R^ |
| pCR-1-1_K110R_f1 | gacagccaaatgtatcttcctgtgag acaagaagtggccagtcc | Cloning of pPp T4 Pgut1-1^N298/K110R^ |
| pCR-1-1_K191R_r1 | cttaactttcttctcagctc taggctttacggatcgatttttg | pPp T4 Pgut1-1^N298/K191R^ |
| pCR-1-1_K191R_f1 | cgatccgtaaagcctaga gctgagaagaaagttaagaaggaac | pPp T4 Pgut1-1^N298/K191R^ |
| pCR-1-1_K197R_r1 | atgggttgaacgttcctttc taactttcttctcagccttaggc | pPp T4 Pgut1-1^N298/K197R^ |
| pCR-1-1_K197R_f1 | ctgagaagaaagttaga aaggaacgttcaacccataatatg | pPp T4 Pgut1-1^N298/K197R^ |

Table S4: K. phaffii strains used during this study.

| **Strain** | **Genotype** | **Source** |
| --- | --- | --- |
| CBS4735 | CBS7435 *his4*Δ | (6) |
| MH458 | CBS7435 *his4*Δ *erg5Δ::P_GAP_--DHCR7-ZeoR erg6Δ::P_GAP_-DHCR24-G418^R^* | (7) |
| yFG046 | CBS 7435 *his4*Δ *hms1-1* | This study |
| yFG051 | CBS 7435 *his4*Δ *hms1-2* | This study |
| yFG060 | CBS 7435 *his4*Δ *hms1-1 hms1-2* | This study |
| yFG071 | CBS 7435 *his4*Δ *hms1-1 hms1-2 his4::P_TEF1-_3HA-HMS1-1-V5* | This study |
| yFG073 | CBS 7435 *his4*Δ *hms1-1 hms1-2 his4::P_TEF1-_3HA-HMS1-2-V5* | This study |
| yFG049 | CBS 7435 *his4*Δ *3HA-HMS1-1* | This study |
| yFG059 | CBS 7435 *his4*Δ *3HA-HMS1-2* | This study |
| yLL109 | CBS 7435 *his4*Δ *P_TEF_-TIR1-FLAG-Zeo^R^* | (4) |
| yMIM073 | CBS 7435 *his4*Δ P_TEF_*-TIR1-FLAG UPC2-AID-STREP* | This study |
| yMIM93 | CBS 7435 *his4*Δ *hms1-1 hms1-2 his4::P_TEF1-_3HA-HMS1-1-V5 upc2* | This study |
| yMIM94 | CBS 7435 *his4*Δ *hms1-1 hms1-2 his4::P_TEF1_ 3HA-HMS1-2-V5 upc2* | This study |
| yLB200 | CBS7435 *his4*Δ *his4::P_TEF2_*-*WSC1*_TMD_-mNG-3FLAG-(HIS)_6_ | (1) |
| yLL108 | CBS 7435 *his4*Δ *P_HTA_-TIR1-FLAG Zeo^R^* | (4) |
| yMIM 83 | CBS7435 *his4*Δ *P_GAP_-SCP-FLAG Zeo^R^* | This study |
| yMIM127 | CBS7435 *his4*Δ *3HA-HMS1-1 P_GAP_-SCP-FLAG Zeo^R^* | This study |
| yMIM128 | CBS7435 *his4*Δ *3HA-HMS1-2 P_GAP_-SCP-FLAG Zeo^R^* | This study |
| yMIM46 | CBS7435 *his4*Δ *scp* | This study |
| yMIM51 | CBS7435 *his4*Δ *hms1-1 scp* | This study |
| yMIM54 | CBS7435 *his4*Δ *hms1-2 scp* | This study |
| yMIM57 | CBS7435 *his4*Δ *hms1-1 hms1-2 scp* | This study |
| yMIM47 | CBS7435 *his4*Δ *dsc1* | This study |
| yMIM50 | CBS7435 *his4*Δ *hms1-1 dsc1* | This study |
| yMIM53 | CBS7435 *his4*Δ *hms1-2 dsc1* | This study |
| yMIM56 | CBS7435 *his4*Δ *hms1-1 hms1-2 dsc1* | This study |
| yMIM59 | CBS7435 *his4*Δ *3HA-HMS1-1 scp* | This study |
| yMIM61 | CBS7435 *his4*Δ *3HA-HMS1-1 dsc1* | This study |
| yMIM65 | CBS7435 *his4*Δ *3HA-HMS1-2 scp* | This study |
| yMIM63 | CBS7435 *his4*Δ *3HA-HMS1-2 dsc1* | This study |
| yMIM122 | CBS7435 *his4*Δ *hms1-1 hms1-2 P_GUT1_-Hms1-1-Zeo^R^* | This study |
| yMIM113 | CBS 7435 *his4*Δ *hms1-1 hms1-2 P_GUT1_-Hms1-1^N268^-Zeo^R^* | This study |
| yMIM117 | CBS 7435 *his4*Δ *hms1-1 hms1-2 P_GUT1_-Hms1-1^N298^-Zeo^R^* | This study |
| yMIM116 | CBS 7435 *his4*Δ *hms1-1 hms1-2 P_GUT1_-Hms1-1^N353^-Zeo^R^* | This study |
| yMIM115 | CBS 7435 *his4*Δ *hms1-1 hms1-2 P_GUT1_-Hms1-1^N400^-Zeo^R^* | This study |
| yMIM121 | CBS 7435 *his4*Δ *hms1-1 hms1-2 P_GUT1_-Hms1-2-Zeo^R^* | This study |
| yMIM118 | CBS 7435 *his4*Δ *hms1-1 hms1-2 P_GUT1_-Hms1-2^N265^-Zeo^R^* | This study |
| yMIM119 | CBS 7435 *his4*Δ *hms1-1 hms1-2 P_GUT1_-Hms1-2^N314^-Zeo^R^* | This study |
| yMIM120 | CBS 7435 *his4*Δ *hms1-1 hms1-2 P_GUT1_-Hms1-2^N344^-Zeo^R^* | This study |
| yMIM131 | CBS 7435 *his4*Δ *hms1-1 hms1-2 P_GUT1_-Hms1-1^N298/K110R^-Zeo^R^* | This study |
| yMIM132 | CBS 7435 *his4*Δ *hms1-1 hms1-2 P_GUT1_-Hms1-1^N298/K191R^-Zeo^R^* | This study |
| yMIM133 | CBS 7435 *his4*Δ *hms1-1 hms1-2 P_GUT1_-Hms1-1^N298/K197R^-Zeo^R^* | This study |

Table S5: Proteins used for in silico analysis during this study.

| **Protein of interest** | **Identifier** |
| --- | --- |
| *Kp* Hms1-1 | CAH2449458.1 |
| *Kp* Hms1-2 | CAH2449479.1 |
| *Ca* Cph2 | [Q59RL7.1](https://www.ncbi.nlm.nih.gov/protein/Q59RL7.1?report=genbank&log$=prottop&blast_rank=1&RID=YGCYRXGM013) |
| *Yl* Sreb | XP_502856.2 |
| *Sp* Sre1 | [Q9UUD1.1](https://www.ncbi.nlm.nih.gov/protein/Q9UUD1.1?report=genbank&log$=protalign&blast_rank=2&RID=YGCT6G7N013) |
| *Sc* Hms1 | [Q12398.1](https://www.ncbi.nlm.nih.gov/protein/Q12398.1?report=genbank&log$=prottop&blast_rank=3&RID=YGCT6G7N013) |
| *Kp* Upc2 | CAH2448785.1 |
| *Ca* Upc2 | XP_711879.1 |
| *Sc* Upc2 | NP_010499.1 |
| *Sc* Emc22 | NP_013329.1 |
| *Cg* Upc2 | 7VPR_A |
| *Kp* Scp | CAH2447051.1 |
| *Yl* Scp | XP_504843.4 |
| *Sp* Scp | O43043 |
| *Hs* Scap | [Q12770.4](https://www.ncbi.nlm.nih.gov/protein/Q12770.4?report=genbank&log$=prottop&blast_rank=6&RID=MK2VHBJX016) |
| *Sp* Dsc1 | O43085 |
| *Kp* Dsc1 | CAH2450293.1 |
| *Af* Dsc1 | B0XQY0 |
| *Sc* Tul1 | P36096 |

Table S6: Primers for qRT-PCR.

| **Target gene** | **Gene locus** | **Forward** | **Reverse** |
| --- | --- | --- | --- |
| *ERG2* | [LT962479.2](https://www.ncbi.nlm.nih.gov/nucleotide/LT962479.2?report=genbank&log$=nuclalign&blast_rank=1&RID=YWRFM6R2016) (1383471 to 1384124) | GCACACTGGTGTTCATTAC | CTGAGAACCAGGGAGATATAC |
| *ERG3* | [LT962476.2](https://www.ncbi.nlm.nih.gov/nucleotide/LT962476.2?report=genbank&log$=nuclalign&blast_rank=1&RID=YWRUANDV013) (2083544 to 2084698) | ACTACACAAGGTGAGCTATC | CATTGACTATAGGGTCTCTGG |
| *ERG4* | [LT962479.2](https://www.ncbi.nlm.nih.gov/nucleotide/LT962479.2?report=genbank&log$=nuclalign&blast_rank=1&RID=YWRWJXB0013) (1661577 to 1663019) | AAAGGGTGAGGAGTTGATAG | GTAGGTATATGGCACTCCAG |
| *ERG5* | [LT962478.2](https://www.ncbi.nlm.nih.gov/nucleotide/LT962478.2?report=genbank&log$=nuclalign&blast_rank=1&RID=YWRZ22XC013) (1269969 to 1271555) | TGACCCAGAAGTGTATGAAA | TGGTCCATTACCAAATACGA |
| *ERG6* | [LT962478.2](https://www.ncbi.nlm.nih.gov/nucleotide/LT962478.2?report=genbank&log$=nuclalign&blast_rank=1&RID=YWS1EVFB016) (1521632 to 1522783) | CCTCTTTCCGGTCAATTAAAG | CAACGGTTTCAGTGGTAAC |
| *ERG11* | [LT962478.2](https://www.ncbi.nlm.nih.gov/nucleotide/LT962478.2?report=genbank&log$=nuclalign&blast_rank=1&RID=YWS4ABRJ013) (383124 to 384671) | AGTCTCCAAAGGTGTTTCT | GTGTTCAAGATGGTTCCTAAC |
| *ERG24* | [LT962476.2](https://www.ncbi.nlm.nih.gov/nucleotide/LT962476.2?report=genbank&log$=nuclalign&blast_rank=1&RID=YWS6RFCM013) (1325497 to 1326816) | TCAATTGAAACCGAAACTGG | CCAACTCCATGCGATTAAC |
| *ERG25* | [LT962478.2](https://www.ncbi.nlm.nih.gov/nucleotide/LT962478.2?report=genbank&log$=nuclalign&blast_rank=1&RID=YWS9952F016) (2127619 to 2128563) | CTCTCCTGTGGGCTTATTT | GGGAAGTCGTAACCAGAAT |
| *ERG26* | [LT962476.2](https://www.ncbi.nlm.nih.gov/nucleotide/LT962476.2?report=genbank&log$=nuclalign&blast_rank=1&RID=YWSBE3PE016) (2535905 to 2536954) | TCACTGCGACTAGATATCAC | AGCCATTCCAGAGTGTATT |
| *ERG27* | [LT962477.2](https://www.ncbi.nlm.nih.gov/nucleotide/LT962477.2?report=genbank&log$=nuclalign&blast_rank=1&RID=YWSDH37F013) (1210944 to 1211975) | GGCACAATATAACAGGATATACG | GCTACATGCACTTCCATATTT |
| *ERG28* | [LT962478.2](https://www.ncbi.nlm.nih.gov/nucleotide/LT962478.2?report=genbank&log$=nuclalign&blast_rank=1&RID=YWSF8K53013) (1197482 to 1197910) | AGCTTACGTTTGCTTCTTATAC | CCTGCTAGCTTACTACCTTT |
| *RSC2* | [LT962477.2](https://www.ncbi.nlm.nih.gov/nucleotide/LT962477.2?report=genbank&log$=nuclalign&blast_rank=1&RID=YWSJJPBK016) (105627 to 108255) | CACTCCTGTCAATAACTA | CTGTGGAGAACGTCAACT |
| *TAF10* | [LT962476.2](https://www.ncbi.nlm.nih.gov/nucleotide/LT962476.2?report=genbank&log$=nuclalign&blast_rank=1&RID=YWSP5AY5013) (1738510 to 1739163) | GAGGCTCCTCACATTTCTATG | GTAACTGCATCGGGAATAATT |

References

1. Bernauer, L., Berzak, P., Lehmayer, L., Messenlehner, J., Oberdorfer, G., Zellnig, G., Wolinski, H., Augustin, C., Baeck, M., and Emmerstorfer-Augustin, A. (2023) Sterol interactions influence the function of Wsc sensors. *J Lipid Res.* **64**, 100466, 10.1016/j.jlr.2023.100466

2. Nishimura, K., Fukagawa, T., Takisawa, H., Kakimoto, T., and Kanemaki, M. (2009) An auxin-based degron system for the rapid depletion of proteins in nonplant cells. *Nat Methods*. **6**, 917–922

3. Ahmad, M., Winkler, C. M., Kolmbauer, M., Pichler, H., Schwab, H., and Emmerstorfer-Augustin, A. (2019) *Pichia pastoris* protease-deficient and auxotrophic strains generated by a novel, user-friendly vector toolbox for gene deletion. *Yeast.* **36**, 557–570

4. Lehmayer, L., Bernauer, L., and Emmerstorfer-Augustin, A. (2022) Applying the auxin-based degron system for the inducible, reversible and complete protein degradation in *Komagataella phaffii*. *iScience.* **25**, 104888, 10.1016/j.isci.2022.104888

5. Radkohl, A., Schusterbauer, V., Bernauer, L., Rechberger, G. N., Wolinski, H., Schittmayer, M., Birner-Gruenberger, R., Thallinger, G. G., Leitner, E., Baeck, M., Pichler, H., and Emmerstorfer-Augustin, A. (2024) Human sterols are overproduced, stored and excreted in yeasts. *Int J Mol Sci.* **25**, 10.3390/ijms25020781

6. Näätsaari, L., Mistlberger, B., Ruth, C., Hajek, T., Hartner, F. S., and Glieder, A. (2012) Deletion of the *Pichia pastoris* *KU70* homologue facilitates platform strain generation for gene expression and synthetic biology. *PLoS One.* **7**, e39720, 10.1371/journal.pone.0039720

7. Hirz, M., Richter, G., Leitner, E., Wriessnegger, T., and Pichler, H. (2013) A novel cholesterol-producing *Pichia pastoris* strain is an ideal host for functional expression of human Na,K-ATPase α3β1 isoform. *Appl Microbiol Biotechnol.* **97**, 9465–9478
